# Carbocisteine or Hypertonic Saline for Acute Respiratory Failure

**DOI:** 10.1056/NEJMoa2603406

**Published:** 2026-06-10

**Authors:** Bronwen Connolly, Naomi Dickson, Christina Campbell, Judy M Bradley, Brenda O’Neill, Ashley Agus, Mike Barker, Jeremy S Bewley, Bronagh Blackwood, Mark Borthwick, Luigi Camporota, Marc Chikhani, Mike Clarke, Paul Dark, Lisa Higgins, Penelope Lambert, Thomas Lunn, Cliona McDowell, Margaret McFarland, Una McShane, Reena Mehta, Ben Messer, Ben Morton, Nehal Patel, Gavin D Perkins, Deborah Rowley, Murali Shyamsundar, Jonathan A Silversides, Gordon Sturmey, John Warburton, Barry Williams, Ranjit Lall, Daniel F McAuley

**Affiliations:** 1 Wellcome-Wolfson Institute for Experimental Medicine, Queen’s University Belfast, Belfast, UK; 2 Department of Physiotherapy, University of Melbourne, Melbourne, Australia; 3 Northern Ireland Clinical Trials Unit, Belfast, UK; 4 Institute of Nursing and Health Research, Ulster University, Belfast, UK; 5 Pharmacy Department, Guys and St Thomas’ NHS Foundation Trust, London, UK; 6 School of Cancer & Pharmaceutical Sciences, Faculty of Life Sciences and Medicine, King’s College London, London, UK; 7 Intensive Care, Bristol Royal Infirmary, University Hospitals Bristol and Weston NHS Foundation Trust, Bristol, UK; 8 Pharmacy Department, Oxford University Hospitals NHS Foundation Trust, Oxford, UK; 9 Critical Care, Guy’s & St Thomas’ NHS Foundation Trust, London, UK; 10 Centre for Human & Applied Physiological Sciences, School of Basic & Medical Biosciences, King’s College London, London, UK; 11 Division of Anaesthetics, Pain Medicine, and Intensive Care, Department of Surgery and Cancer, Imperial College London, London, UK; 12 Critical Care and Anaesthesia, Nottingham University Hospitals NHS Trust, Nottingham, UK; 13 Centre for Public Health, Queen’s University Belfast, Belfast, UK; 14 Division of Immunology, Immunity to Infection and Respiratory Medicine, University of Manchester, Manchester, UK; 15 Northern Care Alliance NHS Foundation Trust (Salford Care Organisation), Salford, UK; 16 Critical Care Unit, University Hospitals of North Midlands, Stoke-on-Trent, UK; 17 Pharmacy Department, Belfast Health and Social Care Trust, Belfast, UK; 18 Pharmacy Department, King’s College Hospital NHS Foundation Trust, London, UK; 19 Department of Critical Care Medicine, King’s College Hospital NHS Foundation Trust, London, UK; 20 Royal Victoria Infirmary, Newcastle upon Tyne Hospitals NHS Foundation Trust, Newcastle upon Tyne, UK; 21 Department of Clinical Sciences, Liverpool School of Tropical Medicine, Liverpool, UK; 22 Department of Critical Care (Aintree site), University Hospitals of Liverpool Group NHS Foundation Trust, Liverpool, UK; 23 Warwick Medical School, University of Warwick, Warwick, UK; 24 Department of Critical Care, Belfast Health and Social Care Trust, Belfast, UK; 25 Independent Patient Partner, UK; 26 Warwick Clinical Trials Unit, University of Warwick, Warwick, UK

## Abstract

**Background:**

Mucoactives are widely used in acute respiratory failure despite limited evidence of effectiveness or safety.

**Methods:**

In this open-label, randomized, full, two-by-two factorial, multicenter trial, critically ill mechanically ventilated adults with acute respiratory failure who had difficult-to-clear secretions were randomly assigned to usual care plus carbocisteine (750 mg three times daily enterally), nebulized hypertonic saline (4ml, 6% or 7% concentration, four times daily), or both interventions, for up to 28 days, or to usual care alone. The primary outcome was duration of mechanical ventilation (time from randomization until first successful unassisted breathing). Primary comparisons were between carbocisteine and no carbocisteine, and hypertonic saline and no hypertonic saline, with each comparison consisting of two groups.

**Results:**

1956 participants were randomized; 486 to carbocisteine (472 analyzed), 485 to hypertonic saline (474 analyzed), 492 to carbocisteine and hypertonic saline (479 analyzed), and 493 to usual care alone (478 analyzed). There was no statistical evidence of treatment interaction (hazard ratio [HR] 1.01, 95% confidence interval [CI] 0.83 to 1.22, P = 0.91). The median duration of mechanical ventilation was 186.1 (95% CI 168.3 to 196.6) hours with carbocisteine and 172.7 (95% CI 165.2 to 190.4) hours with no carbocisteine (adjusted HR [95% CI], 0.96 [0.87 to 1.05; P = 0.345]), and 184.5 (95% CI 165.6 to 194.1) hours with hypertonic saline and 174.3 (95% CI 166.9 to 192.7) hours with no hypertonic saline (adjusted HR [95% CI], 1.00 [0.91 to 1.10]; P = 0.980). Clinically important upper gastrointestinal bleeding occurred significantly more often in participants allocated to carbocisteine (13/965 (1.4%) vs 2/966 (0.2%), risk ratio 6.51 (95% CI 1.47 to 28.76), P = 0.014). Bronchoconstriction requiring nebulized bronchodilators (23/967 (2.4%) vs 4/964 (0.4%), risk ratio 5.73 (95% CI 1.99 to 16.52), P = 0.001) and hypoxemia during nebulization (40/967 (4.1%) vs 3/964 (0.3%), risk ratio 13.29 (95% CI 4.12 to 42.83), P = <0.001) both occurred significantly more often among participants allocated to hypertonic saline. One serious adverse reaction was reported in the combination group (0.2% of patients).

**Conclusions:**

In critically ill patients with acute respiratory failure, neither carbocosteine nor hypertonic saline significantly reduced duration of mechanical ventilation, but were each associated with harm. (Funded by the National Institute for Health and Care Research Health Technology Assessment Programme and others; ISRCTN Registry number, ISRCTN17683568).

## Background

Acute respiratory failure (ARF) is the dominant cause for patient admission to the intensive care unit (ICU).^1–3^ Invasive mechanical ventilation leaves patients predisposed to retained airway secretions secondary to disrupted mucociliary clearance and altered secretion properties (e.g. volume and rheology). To address this, mucoactives are often used in patients with difficult-to-clear secretions.^4,5^ International data report mucoactive use occurring in more than 80% of ICUs,^5,6^ in around 20-30% of patients receiving invasive mechanical ventilation,^6,7^ with two of the most common mucoactives being carbocisteine (a mucoregulator) and hypertonic saline (an expectorant).^5–7^ A systematic review from 2020 concluded the available evidence for use of mucoactives in ARF was limited.^8^ Of note, no data existed to support or refute the use of carbocisteine, and findings for hypertonic saline were inconsistent and low-quality.

Therefore, we conducted a trial to evaluate the clinical effectiveness of carbocisteine and hypertonic saline in addition to usual care compared with usual care alone in adult patients with ARF.

## Methods

### Trial design and oversight

The Mucoactives in Acute Respiratory failure: Carbocisteine and Hypertonic saline (MARCH) trial was a multicenter, full, 2x2 factorial, open-label, randomized phase 3 trial involving critically ill participants with ARF. The trial was conducted at 71 sites across the United Kingdom (Table S1 and S2, Supplementary Appendix, available with the full text of this article at NEJM.org). The protocol (available at NEJM.org) which has been previously published, describes the trial in full.^9^ The authors listed in the protocol designed the study, and there were no important changes after trial commencement.

Data were collected by trial site investigators and analyzed independently by the Northern Ireland Clinical Trials Unit. The first draft of manuscript was written by the lead and senior authors (Connolly, McAuley), all authors critically reviewed drafts, made the decision to submit for publication, vouch for the accuracy and completeness of the data, and adherence to the final protocol and statistical analysis plan. No confidentiality agreements were made that precluded publication of trial findings. The funders (National Institute for Health and Care Research Health Technology Assessment Programme and Belfast Health and Social Care Trust Charitable Trust Fund) had no role in designing the trial, analyzing the data, writing the manuscript, or submitting the manuscript for publication.

The Sponsor of the trial was the Belfast Health and Social Care Trust, and the Chief and Co-Chief Investigators took overall responsibility for trial conduct. The trial was conducted in accordance with the principles of the Declaration of Helsinki, the Good Clinical Practice guidelines of the International Council for Harmonization, and applicable regulatory requirements. Ethical approval was granted on October 28, 2021 by Leeds East (Yorkshire & The Humber) Research Ethics Committee (reference 21/YH/0234). All participants provided written, informed consent via either Personal or Professional Legal Representatives, and subsequently directly when capacity was regained.

### Participants

Eligible participants were adults aged ≥16 years, invasively mechanically ventilated with ARF, with difficult-to-clear secretions according to clinician judgement. Key exclusion criteria were pre-existing chronic respiratory conditions requiring routine mucoactive use, mucoactive treatment commenced more than 24 hours prior to trial enrolment, and known adverse reaction to either carbocisteine or hypertonic saline. Full eligibility criteria are reported in the Supplementary Appendix (Methods S1).

### Randomization

Randomization was performed via an automated web-based or telephone system (Centre for Healthcare Randomized Trials, University of Aberdeen, UK), via randomly permuted blocks in a 1:1:1:1 ratio to the four study groups, and with stratification by site. The randomization sequence was concealed to all but the trial statistician. Participants, treating clinicians, and outcome assessors were aware of treatment assignments.

### Interventions

All participants received usual care and were randomly assigned to carbocisteine, hypertonic saline, carbocisteine and hypertonic saline, or to usual care alone (Supplementary Appendix, Methods S2 and S3). For carbocisteine, the dose was 750 mg three times daily, delivered enterally for up to 28 days. For hypertonic saline, the dose was 4ml, 6% or 7% concentration, delivered via nebulization, four times daily, for up to 28 days. Selection of hypertonic saline concentration was pragmatic, depending on local availability at each study site and reflecting empirical interchangeable use in clinical practice.^4^ Both mucoactives were used according to recommended indications and dosages.^10^

### Outcomes

The primary outcome was the duration of mechanical ventilation (hours) defined as the time from randomization until first successful unassisted breathing (maintaining unassisted breathing at 48 hours) or death.^11^ Unassisted breathing was defined as no inspiratory support or extracorporeal lung support. The duration included time receiving extracorporeal lung support, invasive mechanical ventilation, and non-invasive ventilation delivering volume or pressure support ventilation; and excluded time receiving high-flow oxygen therapy and continuous positive airway pressure. Patients with a tracheostomy/endotracheal tube in situ could still achieve successful unassisted breathing, and follow-up was to 60 days from randomization. Further explanatory details of the primary outcome are reported in the Supplementary Appendix (Methods S4).

Secondary clinical and safety outcomes, timing of their assessment, and measurement tools, are detailed in Table 2 and the Supplementary Appendix (Table S3). Clinical outcomes included extubation, re-intubation, duration of ICU and hospital stay, all-cause mortality, health-related quality of life, respiratory physiotherapy input, and antibiotic usage (for respiratory infections). Safety was measured by the occurrence of four pre-specified outcomes: clinically important upper gastrointestinal bleeding, bronchoconstriction requiring nebulized bronchodilators, ventilator or circuit dysfunction with respiratory deterioration, and hypoxemia during nebulization. Clinical and safety outcomes were measured at baseline and daily up to and including Day 28 (or if the primary outcome was reached), or ICU discharge, or death, whichever came first. Hospital resource use and health-related quality of life data (across hospital, Day 60, and 6-month timepoints) will be reported separately in the pre-planned health economic analysis.

Site investigators reported serious adverse events considered at least possibly related to either of the study mucoactives during the period of administration.

### Statistical analysis

We calculated a sample of size of 1956 (489 in each of the four randomized groups) was required to show a 1 day treatment reduction effect^12^ in duration of mechanical ventilation for each of the intervention groups separately i.e. carbocisteine versus no carbocisteine and hypertonic saline versus no hypertonic saline, based on a median duration of mechanical ventilation of 7 days ^13,14^ The minimum clinically important value of 1 day was also based on discussion with patient and family member advisors, who emphasized the importance of reducing time spent on the ventilator as a priority outcome.^15^ This median duration of mechanical ventilation and 1 day reduction treatment effect resulted in a hazard ratio of 0.86. Based on a log-rank test and at 90% power and a significance level of 0.05, we therefore required a sample of size of 1856 (464 in each of the four randomized groups); allowing a loss to follow-up at the 5% level, required a sample size of 1956 (489 in each of the four randomized groups). As there was no anticipated interaction between the two mucoactives, the sample size was not inflated for this purpose.^16^ Results are reported according to the CONSORT Extension for Factorial Trials.^17,18^

The comparisons were between carbocisteine (patients assigned to receive carbocosteine alone or carbocisteine plus hypertonic saline) and no carbocisteine (patients assigned to receive hypertonic saline alone or usual care), and between hypertonic saline (patients assigned to receive hypertonic saline alone or carbocisteine plus hypertonic saline) and no hypertonic saline (patients assigned to receive carbocisteine alone or usual care). Each comparison was assessed with a type I error rate of 0.05, with no adjustment for multiple testing. No interactions were anticipated between carbocisteine and hypertonic saline; this theory was formally tested before testing for the main comparisons. Descriptive summary measures were used along with tabulation of the amount of missing data and standard approaches to detect patterns in missing data (visual inspection, calculation of proportions, and assessment of the distribution of missing data). Missing data for the primary outcome were rare. Visual inspection and tabulation showed no evidence of differential missingness between treatment groups or across key baseline variables. As a result, all primary analyses were conducted using observed data only. No missing outcome data were imputed. The primary comparisons were assessed using a two-sided significance level of 0.05. Because the SAP did not include a provision for correcting for multiplicity when conducting tests for secondary endpoints or in subgroups, results are reported as point estimates and 95% confidence intervals. The widths of the confidence intervals have not been adjusted for multiplicity and should not be used to infer definitive treatment effects.

The primary analysis was conducted on outcome data from all randomized patients on an intention-to-treat basis. Primary outcome data for the comparison groups were compared using a Cox proportional hazards (Cox-PH) model including site and adjusting for age and illness severity (APACHE II) with hazard ratio (HR) and 95% confidence intervals (CI) presented. There was no evidence of violation of the proportional hazards assumption based on Schoenfeld residual tests, graphical assessment of scaled residuals, or inspection of log–log survival plots for the primary treatment effects. An unadjusted analysis was also conducted. Post hoc pairwise comparisons between treatment groups were also conducted using hazard ratios from Cox proportional hazards models adjusted for age and APACHE II score with a shared frailty for site. A post hoc complier average causal effect (CACE) analysis was conducted to estimate the causal effect of carbocisteine and hypertonic saline among patients who complied with their randomized allocation (Supplementary Appendix, Methods S5).

A secondary analysis of the primary outcome was conducted for survivors (patients extubated or still ventilated prior to day 60) and non-survivors, and we included sensitivity analyses for competing risk of death, impact of contamination, and impact of compliance. As termination of mechanical ventilation may occur for both favorable and poor clinical reasons, the number of deaths in each group was reported. Pre-specified exploratory subgroup analyses were conducted with results presented using estimated treatment effects and 99% CI. These were chosen to provide a conservative representation of uncertainty and to discourage over-interpretation in the context of multiple subgroup comparisons. Evaluation of secondary continuous outcomes used analysis of covariance to adjust for baseline characteristics and covariates such as age, severity of hypoxemia (based on PaO_2_/FiO_2_ ratio) and APACHE II. In addition to the adjusted analyses, unadjusted analyses were also conducted. Evaluation of secondary binary outcomes used generalized linear models as appropriate to estimate risk ratios and 95% CI.

## Results

### Trial population

**Figure 1:**
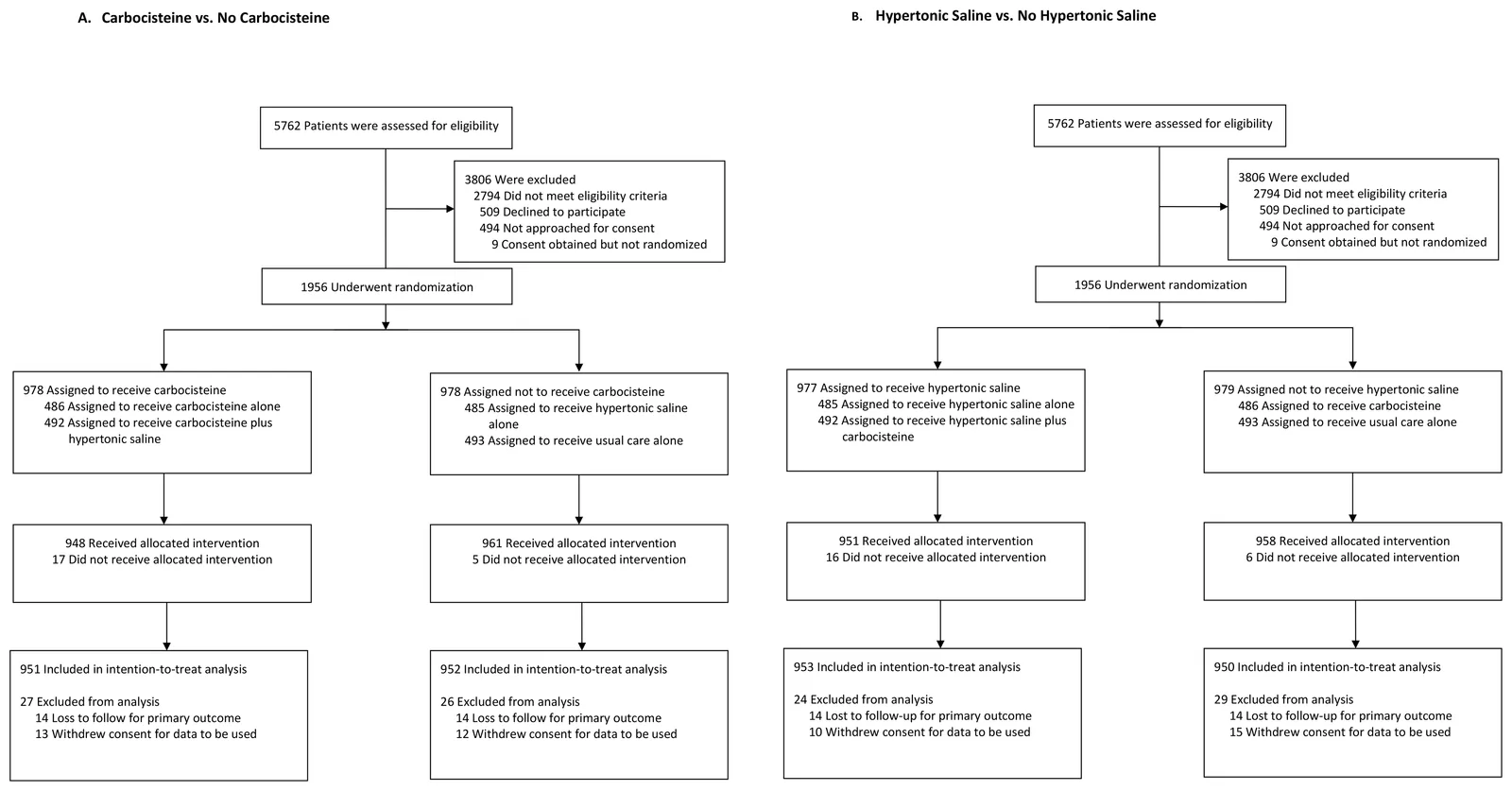
Screening, Randomization, and Assessment Flow diagram of screening, randomization, and assessment of participants by comparison. A. Carbocisteine vs. No Carbocisteine. B. Hypertonic Saline vs. No Hypertonic Saline A flow diagram for each of the four treatment groups is shown in the Supplementary Appendix, Figure S1.

Between February 17, 2022 and April 30, 2025, a total of 1956 participants underwent randomization (Figure 1 and Supplementary Appendix, Figure S1). Follow-up of the last recruited participant was completed on October 31, 2025. Baseline characteristics of participants included in the main comparisons are shown in Table 1 and Table S4 (Supplementary Appendix). Overall, the characteristics at baseline among participants in the comparison groups were similar. There was no evidence of a statistical interaction between carbocisteine and hypertonic saline (interaction HR 1.01, 95% CI 0.83– 1.22; P = 0.91). As pre-specified in the factorial design, this interaction test was performed prior to the primary comparisons to confirm that main-effects analyses were appropriate. Across comparison groups, duration of treatment was approximately 12 days of therapy and treatment compliance was approximately 85% (Supplementary Appendix, Table S5).

### Primary outcome

There was no significant difference in the duration of mechanical ventilation with either carbocisteine or hypertonic saline (Table 2 and Supplementary Appendix, Table S6). The median duration of mechanical ventilation was 186.1 (95% CI, 168.3 to 196.6) hours with carbocisteine and 172.7 (95% CI, 165.2 to 190.4) hours with no carbocisteine (adjusted HR [95% CI], 0.96 [0.87 to 1.05; P = 0.345]), and was 184.5 (95% CI, 165.6 to 194.1) hours with hypertonic saline and 174.3 (95% CI, 166.9 to 192.7) hours with no hypertonic saline (adjusted HR [95% CI], 1.00 [0.91 to 1.10]; P = 0.980). Kaplan-Meier curves for the primary outcome are shown in the Supplementary Appendix (Figure S2). Individual summary estimates of the primary outcome in each of the four treatment groups, and pairwise comparisons, are reported in the Supplementary Appendix (Table S6 and Table 7), and results were similar to the primary analysis.

Results for the post hoc CACE analysis and prespecified secondary analysis are reported in the Supplementary Appendix (Table S8, and Tables S9 and S10 and Figures S3 and S4, for survivors and non-survivors respectively). Results were similar to the primary analysis. Subgroup and sensitivity analyses are reported in the Supplementary Appendix (Table S11 and Table S12 respectively). Deaths at Day 28 in the carbocisteine group were 112 (23.4%), in the hypertonic saline group were 108 (22.5%), in the carbocisteine and hypertonic saline group were 108 (22.2%), and in the usual care group were 121 (25.0%) (Supplementary Appendix, Table S5).

### Secondary outcomes

Results for the secondary clinical outcomes are shown in Table 2.

### Safety outcomes

Clinically important upper gastrointestinal bleeding was uncommon overall (15/1931; 0.8%) but occurred significantly more often in participants who received carbocisteine (13/965; 1.4% vs 2/966; 0.2%), corresponding to a risk ratio of 6.51 (95% CI 1.47 to 28.76, p=0.014)(Table 3). For the other pre-specified safety outcomes there was no difference between participants who received and did not receive carbocisteine (Table 3).

Bronchoconstriction requiring nebulized bronchodilators was uncommon overall (27/1931; 1.4%) but occurred significantly more often among participants who received hypertonic saline (23/967; 2.4% vs 4/964; 0.4%), corresponding to a risk ratio of 5.73 (95% CI 1.99 to 16.52, p=0.001) (Table 3). Similarly, hypoxemia during nebulization was uncommon (43/1931; 2.2%), but also occurred significantly more often among participants who received hypertonic saline (40/967; 4.1% vs 3/964; 0.3%), corresponding to a risk ratio of 13.29 (95% CI 4.12 to 42.83, P = <0.001) (Table 3). Clinically important upper gastrointestinal bleeding or ventilator or circuit dysfunction with respiratory deterioration were not more common in participants who received hypertonic saline (Table 3).

Serious adverse reactions and suspected unexpected serious adverse reactions were rare across all treatment groups. Only one serious adverse reaction was reported in the combination group (0.2% of patients) which was assessed to be expected and related to carbocisteine (a lower gastrointestinal bleed requiring tranexamic acid and blood product transfusion). No unexpected adverse, or suspected unexpected serious adverse events related to study intervention occurred in any group (Supplementary Appendix Table S13).

## Discussion

In this randomized trial, the addition of carbocisteine or hypertonic saline to usual care in critically ill patients with ARF showed no benefit. Both mucoactives were associated with harm, highlighting the need for caution in their use in clinical practice.

Empirically, mucoactives are used to enhance airway clearance in patients receiving invasive mechanical ventilation with difficult-to-clear secretions.^4,5^ Our findings address previous uncertainty regarding the usefulness of two mucoactive therapies in ARF,^8^ by demonstrating lack of clinical effectiveness of carbocisteine and hypertonic saline – two of the most commonly used mucoactives – and underpinning recent observational studies suggesting de-implementation of mucoactives as examples of low-value care.^19,20^ Increased gastrointestinal bleeding was seen with carbocisteine and nebulization-related harms with hypertonic saline, the latter aligning with previous data investigating N-acetylcysteine,^21^ and raise concern for their continued use. Of note, similar findings of clinical ineffectiveness andadverse event reporting (increased number of gastrointestinal disorder events in patients receiving carbocisteine compared to those who do not, and respiratory disorder events in patients receiving hypertonic saline compared to those who do not) are evident in other respiratory diseases such as bronchiectasis.^22^

The trial included a large sample of participants with ARF with high illness acuity and varied diagnostic categories including around 20% of patients with acute respiratory distress syndrome. Beyond the primary analysis, subgroup analyses were uninformative regarding characteristics that could influence underlying secretion load and/or functioning of innate airway clearance mechanisms and in whom mucoactives may be hypothesized to be more beneficial. Further research would be needed to extend investigation of whether some critically ill patients might benefit from mucoactive agents. Understanding potential heterogeneity of treatment effect of carbocisteine and hypertonic saline in ARF would be important to determine if specific characteristics influence outcome,^23^ and future mechanistic analysis could further elucidate this.^24^

A key eligibility criterion for MARCH was the presence of difficult-to-clear secretions. Determination of this was by the clinical team in the absence of any standardized definition and potential inter-clinician variability may exist. Future mechanistic work to develop objective markers of secretion assessment would be valuable for future investigations. We did not collect data on the proportion of patients who received pre-randomization mucoactive therapy and acknowledge that this has the potential to bias our findings towards the null effect. Respiratory physiotherapy, delivering airway clearance techniques, may influence the potential role of mucoactives. Although we did not protocolize, nor collect detailed descriptions of, respiratory physiotherapy for individual participants, occurrence and frequency of airway clearance treatment sessions reflected usual care as previously described.^4^ We rigorously measured pre-specified safety outcomes (in addition to adverse events) across all trial participants based on events previously reported in the literature, ^21,25,26^ which limited the influence of potential reporting bias given the open-label design. We did not undertake a test-dose assessment prior to administration as reported in previous outpatient studies^22^ or protocolize pre-treatment bronchodilator therapy, which may contribute to our findings of harm in patients receiving hypertonic saline. However, these approaches do not reflect current clinical practice in the critically ill, and our exclusion criteria included known adverse reactions to either study intervention.

Despite high levels of compliance in those allocated to both interventions, some participants in the non-intervention groups received carbocisteine or hypertonic saline. This may reflect the limitations of open-label designs and lack of equipoise across all clinicians responsible for the care of trial participants, and interpretation of our findings may be limited by this contamination. That said, our CACE findings supported the primary intention-to-treat analysis. Furthermore, the requirement for blinding and placebo use in clinical trial design is being challenged,^27^ especially where resources required to deliver these features are prohibitive. Importantly our primary outcome, duration of mechanical ventilation, a core trial outcome,^11^ had an objective and standardized definition ensuring consistent measurement. However, we recognize that factors such as sedation practices, extubation thresholds, and other clinical circumstances may still influence this in an open-label trial leading to potential bias and other ventilation-based outcomes may be considered more robust. We recognized duration of mechanical ventilation may be susceptible to bias due to competing risk of death and *a priori* planned a sensitivity analysis for this, as well as followed the core outcome set requirement to report the outcome for survivors and non-survivors separately. The nature of the definition of duration of mechanical ventilation ensured that the primary outcome was evaluable for all randomized patients, including non-survivors, thus avoiding survivor bias. Prioritization of this outcome as primary was also informed by our patient and family member advisors.

MARCH was UK-centric but involved 71 diverse and representative sites. The number of screened and ineligible patients was large albeit related to clear, protocol-defined criteria (e.g. >24-hour prior mucoactive exposure or clinical status); these exclusions were expected and consistent with the target population. The enrolled trial population was representative of national UK critical care admissions^28^. For these reasons, our findings are likely to be considered generalizable. We investigated the two most commonly prescribed mucoactives at the time of study design,^6^ and for which available evidence for effectiveness was either absent (carbocisteine) or uncertain (hypertonic saline).^8^ While the absence of any statistical interaction between the mucoactives supports the factorial design, we recognize that the trial was not formally powered to detect an interaction, is a potential limitation. Although our trial was not powered to exclude clinically important interactions, multiple analyses mitigate concerns regarding violation of the factorial design assumptions. Specifically, pairwise comparisons treating the study as a four-arm trial demonstrated consistent estimates across groups, and there was no evidence of benefit in any individual or combination arm. Our data did not suggest dilution of treatment effects due to lack of mechanism independence of the two interventions. Overall, these findings suggest that the absence of treatment effect is unlikely to be explained by undetected interaction or dilution of effects due to mechanistic overlap. We had minimal (<3%) loss of primary outcome data in our intention-to-treat analysis due to missing data and withdrawal of consent.

In conclusion, in critically ill patients with ARF, neither carbocosteine nor hypertonic saline significantly reduced the duration of mechanical ventilation, but were each associated with harm.

Disclosure forms provided by the authors are available with the full text of this article at NEJM.org.

## Supplementary Material

supplement

## Figures and Tables

**Table 1:** Characteristics of the Participants at Baseline

Characteristic*	Carbocisteine (N=965)	No carbocisteine (N=966)	Hypertonic saline (N=967)	No hypertonic saline (N=964)
**Age (years)**	56.7±15.4	57.5±14.5	56.6±15.1	57.7±14.8
**Sex**				
- **Male**	667 (69.1)	682 (70.6)	678 (70.1)	671 (69.6)
- **Female**	298 (30.9)	284 (29.4)	289 (29.9)	293 (30.4)
**Race or ethnic group^1^**				
- **White**	832 (86.2)	837 (86.7)	831 (85.9)	838 (86.9)
- **Black**	19 (2.0)	26 (2.7)	23 (2.4)	22 (2.3)
- **Asian**	29 (3.0)	28 (2.9)	26 (2.7)	31 (3.2)
- **Unknown**	83 (8.6)	71 (7.4)	83 (8.6)	71 (7.4)
- **Other**	2 (0.2)	4 (0.4)	4 (0.4)	2 (0.2)
**Functional Comorbidity Index^2^**	1.6±1.4	1.6±1.4	1.6±1.4	1.6±1.4
**ICU admission diagnostic category^3^**				
- **Respiratory system**	290 (30.0)	261 (27.0)	284 (29.4)	267 (27.7)
- **Central nervous system**	148 (15.3)	191 (19.8)	158 (16.3)	181 (18.8)
- **Cardiovascular system**	165 (17.1)	153 (15.8)	149 (15.4)	169 (17.5)
**Chronic respiratory condition**	198 (20.5)	212 (22.0)	217 (22.4)	193 (20.0)
**Neurological diagnosis**	240 (24.9)	256 (26.5)	242 (25.0)	254 (26.4)
**Currently receiving antibiotics to treat a pulmonary infection?**	689 (71.4)	693 (71.7)	686 (70.9)	696 (72.2)
**Presence of ARDS**	202 (21.0)	204 (21.2)	206 (21.4)	200 (20.8)
**APACHE II Score^3^**	17.2±6.8 n=964	17.0±6.3 n=966	17.2±6.6 n=967	17.0±6.5 n=963
**SOFA score^4^**	9.3±3.6 n=789	9.4±3.5 n=800	9.4±3.6 n=793	9.3±3.5 n=793
**Tidal Volume (ml)**	470.6±150.8 n=952	466.2±146.5 n=952	467.7±149.7 n=954	469.1±147.7 n=950
**Total Respiratory Rate**	20.7±5.9 n=961	20.8±5.9 n=963	20.7±5.9 n=965	20.8±5.9 n=959
**PEEP (cmH_2_O)**	8.2±2.7 n=956	8.3±2.8 n=954	8.3±2.8 n=957	8.2±2.7 n=953
**PaO_2_/FiO_2_ ratio (mmHg)**	221.1±87.5 n=953	224.0±97.8 n=957	224.6±96.1 n=957	220.4±89.3 n=953
**PaCO_2_ (kPa)**	5.8±1.3 n=954	5.8±1.4 n=956	5.8±1.4 n=958	5.8±1.3 n=952
**pH**	7.40±0.08 n=953	7.40±0.08 n=960	7.41±0.08 n=960	7.40±0.08 n=953
**Type of humidification**				
- **Heated humidification**	694 (78.3)	687 (75.7)	698 (77.6%)	683 (76.4%)
- **Heat moisture exchange**	189 (21.3)	214 (23.6)	199 (22.1%)	204 (22.8%)
- **Other**	3 (0.3)	6 (0.7)	2 (0.2%)	7 (0.8%)

* Data are presented as mean±standard deviation, or n (%). Percentages may not total 100 due to rounding Abbreviations: ICU = intensive care unit, ARDS = Acute Respiratory Distress Syndrome, APACHE = Acute Physiology and Chronic Health Evaluation, SOFA = Sequential Organ Failure Assessment, ^6^PEEP = positive end expiratory pressure, PaO_2_/FiO_2_ = partial pressure of arterial oxygen/fraction of inspired oxygen, PaCO_2_ = partial pressure of arterial carbon dioxide

1 Race or ethnic group data were collected from medical records of participants

2 Functional Comorbidity Index is a 0–18 point scale designed to predict physical function by totaling 18 specific, self-reported, or chart-abstracted chronic conditions (e.g., arthritis, osteoporosis, obesity, diabetes). If present, each condition is assigned 1 point, with higher total scores indicating greater impairment in physical function

3 Full details on all ICU admission diagnostic categories are reported in Supplementary Appendix Table S4

3 Scores on the APACHE II scale range from 0 to 71, with higher scores indicating greater severity of illness

4 Scores on the SOFA scale range from 0 to 24, with higher scores indicating greater severity of disease

**Table 2:** Primary and Secondary Outcomes

Outcome	Carbocisteine Median (95% CI)	No carbocisteine Median (95% CI)	Carbocisteine Vs No carbocisteine (95% CI)	Hypertonic saline Median (95% CI)	No Hypertonic saline Median (95% CI)	Hypertonic saline Vs No Hypertonic saline (95% CI)
**Primary outcome:** **Duration of mechanical** **ventilation (hours)^a^**	186.1 (168.3 to 196.6) (n=951)	172.7 (165.2 to 190.4) (n=952)	0.96 (0.87 to 1.05) P = 0.345	184.5 (165.6 to 194.1) (n=953)	174.3 (165.6 to 194.1) (n=950)	1.00 (0.91 to 1.10) P = 0.980
Secondary outcomes*~						
Time to first successful extubation (hours)^a^^	352.4 (283.1 to 428.2) (n=955)	356.9 (287.3 to 448.6) (n=953)	1.03 (0.92 to 1.15)	403.0 (308.7 to 455.2) (n=958)	321.5 (262.8 to 402.9) (n=950)	1.00 (0.89 to 1.11)
Re-intubation (n, %)^b^	50 (5.2) (n=965)	49 (5.1) (n=966)	1.02 (0.70 to 1.50)	56 (5.8) (n=967)	43 (4.5) (n=964)	1.30 (0.88 to 1.91)
Respiratory physiotherapy input occurrence (n, %)^b^#	955 (99.0) (n=965)	951 (98.5) (n=966)	1.01 (1.00 to 1.02)	953 (98.6) (n=967)	953 (98.9) (n=964)	1.00 (0.91 to 1.10)
Respiratory physiotherapy input frequency (n sessions)^c^^#^	8.0 (7.7 to 9.0) (n=955)	8.0 (8.0 to 9.0) (n=951)	-0.17 (-1.07 to 0.72)	8.0 (7.0 to 9.0) (n=953)	8.0 (8.0 to 9.0) (n=953)	0.04 (-0.86 to 0.94)
Antibiotic usage (number of days)^c^	9.0 (8.0 to 9.0) (n=749)	8.0 (8.0 to 9.0) (n=758)	-0.68 (-1.77 to 0.41)	9.0 (8.0 to 9.0) (n=746)	8.0 (8.0 to 90) (n=761)	-0.21 (-1.31 to 0.88)
Duration of ICU stay (days)^c^^,^^d^	14.0 (13.0 to 15.0) (n=951)	14.0 (13.0 to 15.0) (n=949)	1.01 (0.92 to 1.11)	14.0 (13.0 to 15.0) (n=955)	14.0 (13.0 to 15.0) (n=945)	1.04 (0.95 to 1.14)
Duration hospital stay (days)^c^^,^^d^	29.0 (27.0 to 31.0) (n=929)	27.0 (25.0, 29.0) (n=922)	1.03 (0.91 to 1.17)	29.0 (26.0 to 31.0) (n=928)	28.0 (26.0 to 30.0) (n=923)	1.04 (0.94 to 1.14)
All-cause mortality (Day 28) (n, %)^b^	220 (22.8) (n=965)	229 (23.7) (n=966)	0.96 (0.82 to 1.13)	216 (22.3) (n=967)	233 (24.2) (n=964)	0.92 (0.79 to 1.09)
All-cause mortality (Day 60) (n, %)^b^	245 (25.4) (n=965)	269 (27.9) (n=966)	0.91 (0.79 to 1.06)	248 (25.7) (n=967)	266 (27.6) (n=964)	0.93 (0.80 to 1.08)
All-cause mortality (6 month) (n, %)^b^	276 (28.6) (n=965)	292 (30.2) (n=966)	0.95 (0.82 to 1.09)	281 (29.1) (n=967)	287 (29.8) (n=964)	0.98 (0.85 to 1.12)

a Difference, Hazard ratio (95% confidence interval, CI);

b Difference, Risk ratio (95% CI);

c Difference, Median difference (95% CI);

d Post hoc sub-hazard ratio (SHR) with 95% confidence interval estimated using Fine–Gray competing-risks model adjusted for age and APACHE II including site with cluster robust standard error (SE)

* The widths of confidence intervals have not been adjusted for multiplicity and cannot be used to infer treatment effects

~ Definitions of secondary outcomes: Extubation = Time from randomization to first successful extubation (success defined as remaining free from endotracheal or tracheostomy tubes at 48 hours i.e. those patients with a tracheostomy in situ needed to be decannulated to be considered extubated, and remain decannulated at 48 hours for this to be considered successful) - Censored at 60 days; Re-intubation = Event of reintubation of endotracheal tube after a planned extubation, Excludes temporary reinsertion of endotracheal tube for procedures only - Censored at 60 days; Respiratory physiotherapy input = occurrence and frequency of airway clearance sessions - Censored at Day 28 (or the primary outcome is reached), or ICU discharge, or death, whichever comes first; Antibiotic usage = Dose of individual agents Censored at Day 28 (or the primary outcome is reached), or ICU discharge, or death, whichever comes first; Duration of ICU and hospital stay = Time from randomization until patient first leaves the relevant facility or dies - Censored at 6 months; All-cause mortality = Confirmation and cause of death

^ Total number of patients with tracheostomy, n=701

# Respiratory physiotherapy input relates to airway clearance techniques delivered by respiratory physiotherapists e.g. manual techniques (chest vibration and percussion), positioning/postural drainage, assisted cough. This is distinct from (and does not refer to) other therapy such as nebulized treatments or assessment of readiness/tolerance of spontaneous breathing trials

**Table 3:** Safety Outcomes

Clinically important upper GI bleeding due to peptic ulceration confirmed on upper GI endoscopy*		Hypertonic saline		Total
		No	Yes	
**Carbocisteine**	**No**	2 (0.4%) N=485 (Usual care)	0 (0.0%) N=481 (Hypertonic saline)	2 (0.2%) N=966 (No Carbocisteine)
	**Yes**	7 (1.5%) N=479 (Carbocisteine)	6 (1.2%) N=486 (Hypertonic saline plus Carbocisteine)	13 (1.4%) N=965 (Carbocisteine)
**Total**		9 (0.9%) N=964 (no Hypertonic saline)	6 (0.6%) N=967 (Hypertonic saline)	

	**Carbocisteine**	**No Carbocisteine**	**Hypertonic Saline**	**No Hypertonic Saline**
	13 (1.4%) N=965 (Carbocisteine)	2 (0.2%) N=966 (No Carbocisteine)	6 (0.6%) N=967 (Hypertonic saline)	9 (0.9%) N=964 (No Hypertonic saline)
**Risk Ratio (95% CI)**	6.51 (1.47 to 28.76)		0.66 (0.24 to 1.86)	
**p-value from GLM^a^**	0.014		0.437	

Bronchoconstriction requiring nebulized bronchodilators*		**Hypertonic saline**		**Total**
		**No**	**Yes**	
**Carbocisteine**	**No**	2 (0.4%) N=485 (Usual care)	12 (2.5%) N=481 (Hypertonic saline)	14 (1.5%) N=966 (No Carbocisteine)
	**Yes**	2 (0.4%) N=479 (Carbocisteine)	11 (2.3%) N=486 (Hypertonic saline plus Carbocisteine)	13 (1.4%) N=965 (Carbocisteine)
**Total**		4 (0.4%) N=964 (no Hypertonic saline)	23 (2.4%) N=967 (Hypertonic saline)	

	**Carbocisteine**	**No Carbocisteine**	**Hypertonic Saline**	**No Hypertonic Saline**
	13 (1.4%) N=965 (Carbocisteine)	14 (1.5%) N=966 (No Carbocisteine)	23 (2.4%) N=967 (Hypertonic saline)	4 (0.4%) N=964 (no Hypertonic saline)
**Risk Ratio (95% CI)**	0.93 (0.44 to 2.00)		5.73 (1.99 to 16.52)	
**p-value from GLM^a^**	0.849		0.001	

Ventilator or circuit dysfunction with respiratory deterioration*		**Hypertonic saline**		**Total**
		**No**	**Yes**	
**Carbocisteine**	**No**	4 (0.8%) N=485 (Usual care)	6 (1.3%) N=481 (Hypertonic saline)	10 (1.0%) N=966 (No Carbocisteine)
	**Yes**	2 (0.4%) N=479 (Carbocisteine)	6 (1.2%) N=486 (Hypertonic saline plus Carbocisteine)	8 (0.8%) N=965 (Carbocisteine)
**Total**		6 (0.6%) N=964 (no Hypertonic saline)	12 (1.2%) N=967 (Hypertonic saline)	

	**Carbocisteine**	**No Carbocisteine**	**Hypertonic Saline**	**No Hypertonic Saline**
	8 (0.8%) N=965 (Carbocisteine)	10 (1.0%) N=966 (No Carbocisteine)	12 (1.2%) N=967 (Hypertonic saline)	6 (0.6%) N=964 (no Hypertonic saline)
**Risk Ratio (95% CI)**	0.80 (0.32 to 2.02)		1.99 (0.75 to 5.29)	
**p-value from GLM^a^**	0.638		0.166	

Hypoxemia during nebulization*		**Hypertonic saline**		**Total**
		**No**	**Yes**	
**Carbocisteine**	**No**	2 (0.4%) n=485 (Usual care)	20 (4.2%) n=481 (Hypertonic saline)	22 (2.3%) n=966 (No Carbocisteine)
	**Yes**	1 (0.2%) n=479 (Carbocisteine)	20 (4.1%) n=486 (Hypertonic saline plus Carbocisteine)	21 (2.2%) n=965 (Carbocisteine)
**Total**		3 (0.3%) n=964 (no Hypertonic saline)	40 (4.1%) n=967 (Hypertonic saline)	

	**Carbocisteine**	**No Carbocisteine**	**Hypertonic Saline**	**No Hypertonic Saline**
	21 (2.2%) n=965 (Carbocisteine)	22 (2.3%) n=966 (No Carbocisteine)	40 (4.1%) n=967 (Hypertonic saline)	3 (0.3%) n =964 (no Hypertonic saline)
**Risk Ratio (95% CI)**	0.96 (0.53 to 1.73)		13.29 (4.12 to 42.83)	
**p-value from GLM^a^**	0.880		<0.001	

a using binomial family and log link;

GI = gastrointestinal, GLM = Generalized Linear Model, CI = Confidence Interval

* The widths of confidence intervals have not been adjusted for multiplicity and cannot be used to infer treatment effects
