# Supplementary material for: Carbocisteine or Hypertonic Saline for Acute Respiratory Failure
Source: N Engl J Med. Author manuscript; Available in PMC 2026 Nov 2. (PMC7619674; doi:10.1056/NEJMoa2603406)
Supplement: Supplementary file 1 [file EMS214051-supplement-supplement.pdf]

Carbocisteine or hypertonic saline for acute respiratory failure

SUPPLEMENTARY APPENDIX

Table of contents

MARCH Investigators 2

SUPPLEMENTARY METHODS

S1. Eligibility criteria 15

S2. Usual care and mucoactive interventions 16

S3. Criteria for discontinuing or modifying allocated interventions 17

S4. Clarifications and working examples for the primary outcome 18

S5. Complier Average Causal Effect analysis 21

SUPPLEMENTARY FIGURES

Figure S1. CONSORT flow diagram by treatment group 22

Figure S2. Kaplan-Meier curves: Primary outcome (Intention-to-treat population) 23

Figure S3. Kaplan-Meier curves for duration of mechanical ventilation (survivors) 24

Figure S4. Kaplan-Meier curves: Primary outcome (non-survivors) 25

SUPPLEMENTARY TABLES

Table S1. Site recruitment summary 26

Table S2. Representativeness of Study Participants and Sites 28

Table S3. Detail of secondary outcomes 30

Table S4. Additional characteristic data of participants at baseline 32

Table S5. Treatment after trial entry by comparison group 35

Table S6. Primary outcome (intention to treat) 37

Table S7. Post hoc pairwise comparisons between treatment groups 38

Table S8. Complier Average Causal Effect analysis 39

Table S9. Primary outcome secondary analysis (Survivors) 40

Table S10. Primary outcome secondary analysis (non-survivors) 41

Table S11. Primary outcome subgroup analyses 42

Table S12. Primary outcome sensitivity analyses 45

Table S13. Adverse event reporting 46

REFERENCES **47**

MARCH Investigators

Aberdeen Royal Infirmary, Aberdeen, Scotland

Callum Kaye (Principal Investigator, PI), Abduliah Abdelaty (Physician), Gavin Bailie (Physician), Asimina (Menia) Chairetaki (Pharmacist), Alice Coulson (Advanced Critical Care Practitioner), Emily Ewing (Research Nurse), Rory Gibson (Physician), James MacBrayne (Physician), Felicity Savage (Research Nurse), Teresa Scott (Research Nurse), Kevin Sim (Physician), Joanne Taylor (Research Nurse), Erin Trumper (Research Nurse), Stephanie Watson (Physiotherapist), Stuart Williams (Physician)

Addenbrookes Hospital, Cambridge, England

Charlotte Summers (PI), Katherine Bongaerts (Pharmacist), Cristina Bravo Elvira (Research Nurse), Galina Maneva (Physician), Jocelyn Marshall (Research Nurse), Quan Ooi (Physician), Sam Petty (Consultant Physiotherapist), Petra Polgarova (Research Nurse), Shibu Sasidharan (Physician), Susan Stevenson (Physician)

Aintree Hospital, Liverpool, England

Ben Morton (PI), Barbara Borgatta (Consultant), Prerak Dwivedi (Associate PI), Fred Flyes (Associate PI), Jacqueline Jeffs-Watts (Physiotherapist), Ben Jones (Associate PI), William McCaig (Research Nurse), Michaela McGowan (Research Nurse), Harriet Murrant (Research Nurse), Amie Reddy (Research Nurse), Clare Sales (Pharmacist), Leanne Smith (Research Nurse), Ian Turner-Bone (Research Nurse), Emma Waring (Physiotherapist), Laura Wilding (Research Nurse)

Altnagelvin Hospital, Londonderry, Northern Ireland

Adrian Donnelly (PI), Susan Baird (Physician), Avril Calvert (Physiotherapist), Michelle Fallon (Consultant), Simon Galbraith (Pharmacist), Dympna Houston (Physiotherapist), Edmund McCallion (Pharmacist), Sinead O'Kane (Research Nurse), Breda Robinson (Pharmacy Technician)

Antrim Area Hospital, Antrim, Northern Ireland

Paul Johnston (PI), Rebecca Gibson (Doctor), Ellen Gorman (Clinical Research Fellow), Natalie Hanley (Research Nurse), Michael Matthews (Research Nurse), Jane McGrath (Pharmacist), Shirley McKenna (Lead Pharmacist)

Barnsley Hospital, Barnsley, England

Kenneth Inweregbu (PI), Katie Bacon (Physiotherapist), Abigail Crew (Clinical Trial Assistant), Sarah Cutts (Research Nurse), Claire Frankland (Physiotherapist), Anna Galvin (Research Nurse), Rachel Hewitt (Physiotherapist), Steven Lobaz (Physician), Marta Malaj (Physician), Katharine Miller (Research Nurse), Catherine Mitchell (Physiotherapist), Alice Nicholson (Research Nurse), Kerry Robinson (Lead Technician Clinical Trials), Neermala Sanassee (Pharmacy), Emma Stoner (Research Nurse), Gillian Turrell (Pharmacist), Stephen Wyatt (Physician)

Belfast City Hospital and Royal Victoria Hospital, Belfast, Northern Ireland

Murali Shyamsundar (PI), Matthew Adams (Clinical Trial Administrator), Judit Barabas (Research Technician), Emma Bohan (Research Nurse), Erin Collins (Research Nurse), John Conlon (Research Technician), Jake Deeney (Physiotherapist), Patrick Devlin (Research Administrator), Rhian Dowding (Clinical Trial Administrator), Stephanie Finn (Research Nurse), Nerielle Fundano (Clinical Research Fellow), Ellen Gorman (Clinical Research Fellow), Jackie Green (Research Nurse), Michelle Growcott (Research Administrator), Kirsty Harris (Physiotherapist), Lyndsey Henning (Physiotherapist), Kirsty Jerrard (Physiotherapist), Anna Knox (Physiotherapist), Elliott Lonsdale (Clinical Research Fellow), Victoria Lyons (Physiotherapist), Katherine Martin (Physiotherapist), Colleen McAllister (Physiotherapist), Danny McAuley (Co-Investigator), Julie McAuley (Research Nurse), Margaret McFarland (Pharmacist), Peter McGuigan (Co-Investigator), Laura Moffett Crooks (Physiotherapist),

Ollie Montgomery (Physiotherapist), Andrew Moorehead (Physiotherapist), Niamh O'Hare (Physiotherapist), Aisling O'Neill (Research Nurse), Charlotte Quinn (Research Nurse), Anna Rea (Clinical Trial Administrator), Kiran Reddy (Clinical Research Fellow), Fiona Rodgers (Physiotherapist), Jon Silversides (Co-Investigator), Jill Stephens (Physiotherapist), Christine Turley (Research Nurse), Kathryn Ward (Research Nurse), Brian Wells (Pharmacist), John Whittaker (Physiotherapist), Oran Wilson (Physiotherapist), Chris Wright (Administrator/Data Manager)

Bedford Hospital, Bedford, England

Ana Vochin (PI), Ajibade Adewuyi (Physician), Caroline Clapham (Pharmacist), Carina Galpin (Research Officer), Anne Marie King (Physiotherapy Team Lead), Gbiadesey Kotomah (Physician), Petula Lemessy (Research Nurse), Rachel Lorusso (Clinical Research Nurse), Juliet Morris (Pharmacy), Melchizedek Penacerrada (Lead Research Nurse), Alexandra Svetik (Physician), Tadeh Tahmasi (Lead Trials Pharmacist), Matthew Thomas (Associate PI), Abhay Vaidya (Consultant), Retno Wulandari (Research Practitioner), Omar Zeb (Pharmacist)

Bristol Royal Infirmary, Bristol, England

Jeremy Bewley (PI), Josephine Bonnici (Clinical Trial Coordinator), Susan Calvert (Physiotherapist), Maria Fernanda Londono Castrillion (Research Nurse), Christina Coleman (Research Nurse), Kathleen Corcoran (Research Nurse), Eleanor Daniel (Physiotherapist), Harriet Davies (Research Nurse), Abbie Dell (Trial Co-Ordinator), Georgia Efford (Research Nurse), Lucy Evans (Pharmacist), Carla Farris (Research Nurse), Zoe Garland (Research Nurse), Lisa Grimmer (Research Nurse), Daniel Hill (Research Nurse), Anna Jones (Research Nurse), Angeliki Kolovou (Research Nurse), Oluwatosin Komolafe (Research Nurse), Susan Thomas Koshy (Research Nurse), Casandra Bazan Lacerot (Research Nurse), Penny Lambert (Research Nurse), Ya-Hui Liang (Pharmacist), Thomas Lunn (Physiotherapist), Liz McCullagh (Pharmacist), Eve Maria Hernandez Morano (Research Nurse), Linda Pipira (Trial Administrator), Rachel Shiel (Research Nurse), Katie Sweet (Research Nurse), Philip Tankpara (Research Nurse), John Warburton (Pharmacist), Denise Webster (Research Nurse), Kim Wright (Research Nurse)

Chesterfield Royal Hospital, Chesterfield, England

Sarah Beavis (PI), Hannah Blagnys (Consultant), Karen Campbell (Advanced Clinical Practitioner), Abigail Forsyth (Physician), Conrad Foster (Advanced Clinical Practitioner), Claire Hirst (Consultant), Brett Jackelman (Physician), Adam Mendelski (Research Nurse), Natalie Minskip (Advanced Clinical Practitioner), Emma Moakes (Research Nurse), Kerry Sharp (Advanced Clinical Practitioner), Rheanna Oades (Research Nurse), James Watson (Advanced Clinical Practitioner), Jennifer Wilson (Physiotherapist), Nerys Winnard (Pharmacist)

Derriford Hospital, Plymouth, England

Deborah Webster (PI), Colin Bigham (Consultant), Cianne Bridgland (Research Nurse), Alex Coombs (Consultant), Stephen Copplestone (Consultant), Robert Jackson (Consultant), Julian Lentaigne (Consultant), Lai Fai (Ruth) Mak (Advanced Specialist Pharmacist), Mike Marner (Pharmacy Trial Manager), Daniel Martin (Consultant), Helen McMillan (Advanced Critical Care Practitioner), Nikitas Nikitas (Consultant), Ian O Brien (Consultant), Ana Lucia (Lucy) Sanchez Rodriguez (Research Nurse), Holly Schofield (Research Nurse), Kayleigh Spencer (Research Nurse), Liana Stapleton (Lankester) (Research Nurse), Bethan Stowe (Research Nurse), Jessica Taylor (Lead Physiotherapist), Sam Waddy (Consultant), Natasha Webb (Research Administrator), Jessica Welbourne (Consultant), Ash Williams (Consultant)

Freeman Hospital and Royal Victoria Infirmary Hospital, Newcastle Upon Tyne, England

Ben Messer (PI), Rikzing Bhutia (Physician), Ben Brown (Data Manager and Clinical Trials Coordinator), Lauren Butler (Research Nurse), Jade Deighton (Pharmacist), Leigh Dunn (Research Nurse), Pamela

Garcia (Research Nurse), Luzgie Gavina (Research Nurse), Arti Gulati (Clinical Trials Coordinator), Stephen Hamilton (Physician), Lisa Mageean (Research Nurse), Iain McCullagh (Physician), Josh Nelson (Physician), Aaron Richards (Data Manager), Helen Sanger (Physiotherapist), Ian Storey (Data Manager), Jake Taylor (Physician), Susan Taylor (Research Nurse), Amaan Ul-Hag (Data Manager), Tess Wilkinson (Research Nurse)

Glan Clywd Hospital, Rhyl, Wales

Richard Pugh (PI), Delyth Davies (Rees) (Research Nurse), Kerry Angus (Advance Critical Care Practitioner), Harry Bamber (Physician), Thomas Beach (Consultant), Craig Beaton, (Consultant), Joriling Collins (Physician), Hannah Davies (Research Administrator), Jennifer Davies (Clinical Research Nurse), Andrew Evans (Consultant), Rachael Farnell (Research Administrator), Esther Garrod (Research Nurse), John Glen (Consultant), Remil Ignatius (Clinical Fellow), Virad Kisan (Physician), Caroline Lees (Research Administrator), Rachel Manley (Research Nurse), Hannah Marshall-Nichols (Physiotherapist/Advance Critical Care Practitioner), Claire McGregor (Specialist Pharmacy Technician), Stephanie Rees (Physician), Jamie Scriven (Physician), Venkat Sundaram (Consultant), Andrew Temperton (Physician), George Woods, (Clinical Fellow)

Glasgow Royal Infirmary, Glasgow, Scotland

Kathryn Puxty (PI), Katherine Brown (Physician), Lucy Bullimore (Research Nurse), Susanne Cathcart (Research Nurse), Oi-Yan (Joyce) Chan (Research Nurse), Fiona Christie (Physician), Richard Cowan (Physician), Jenny Cuthill (Physician), Gemma Duff (Research Nurse), Joe Hawry (Physician), Martin Hughes (Physician), Andy Hurst (Research Nurse), Christopher McGovern (Physician), Lorraine McGregor (Research Nurse), Pamela MacTavish (Pharmacy), Maria Nygren (Pharmacy), Lia Paton (Physician), Amy Prance-Smith (Research Nurse), Alex Puxty (Physician), Tara Quasim (Physician), Gemma Riddler (Research Nurse), Dominic Rimmer (Research Nurse), Gary Rogers (Physician), Jennifer Tait (Pharmacy), Callum Taylor (Physician)

Gloucestershire Royal Hospital, Gloucester, England

Marcin Pachucki (PI), Nicholas Dodds (PI), Gemma Baldwin (Research Nurse), Maddie Benson (Research Co-Ordinator), Michael Castillo (Research Nurse), Mark Charania (Physiotherapist), Rachel Durrant (Pharmacist), Holly Farr (Pharmacist), Tracey Franks (Physiotherapist), Jen Griffiths (Research Data Officer), Anne Kendall (Registrar), Hayley Mumford (Research Nurse), Claire Peters (Specialist Physiotherapist), Ashleigh Pritchard (Research Coordinator), Haripriya Rayasam (Clinical Fellow), Leanne Raybould (Research Assistant), Anna Roberts (Clinician), Teresa Tarling (Senior Research Nurse), Rebecca Da Silva Teixeira (Research Co-Ordinator), Nick Vallotton (Research Nurse), Carys Whitby (Research Nurse), David Windsor (Consultant), Stuart Winearls (Clinician)

Golden Jubilee University National Hospital, Clydebank, Scotland

Philip McCall (PI), Christine Aitken (Research Nurse), Jocelyn Barr (Project Assistant), Elizabeth Boyd (Research Nurse), Shelagh Brown (Physiotherapist), Julie Buckley (Research Nurse), Clare Burke (Physiotherapist), Patricia Griffin (Research Nurse), Charlene Hamilton (Research Nurse), Ilkin Kasmani (Pharmacy), Alfie Lloyd (Physician), Douglas Malcolmson (Pharmacy), Rhiannon McAreavey (Physician), Benjamin Shelley (Physician), Keenan Smith (Physician), Kathryn Valdeavella (Research Nurse)

Grange University Hospital, Cwmbrân, Wales

Christie James (PI/Pharmacist), Ifor Capel (Physician), Amy Cardwell (Research Fellow), Shiney Cherian (Research Nurse), Sara Davies (Physiotherapist), Bradley Dawes (Physician), Nick Edwards (Administrator), Ben Eldridge (Physician), Lowri Evans (Physician), Kirsty Foster (Physiotherapist), Anne Frawley (Physician), Nancy Hawkings (Research Nurse), Jonas Hayes (Physician), Oliver Hodgkinson (Physician), Ffion Jones (Research Nurse), Louise Parfitt (Research Nurse), Emily Phillips

(Advance Critical Care Practitioner), Regina Rayes (Research Nurse), Tamas Szakmany (Physician), Jemma Tuffney (Clinical Trial Coordinator)

Guy's and St Thomas' NHS Foundation Trust, London, England

Luigi Camporota (PI), Beatriz Amaro de Jesus (Research Nurse), Mike Barker (Associate PI/Pharmacist), Nick Barrett (Consultant), Aneta Bociek (Research Nurse), Benjie Cendreda (Research Nurse), Fabiola D'Amato (Research Nurse), Carolin Engelhard (Research Administrator), Sarah Fordyce (Research Nurse), Guy Glover (Consultant), Ellie Hendrie (Research Nurse), Eirini Kosifidou (Research Nurse), Rosario Lim (Research Nurse), Moncy Mathew (Pharmacist), Tahmina Matin (Research Administrator), Christopher Meddings (Pharmacist), Vladimir Milic (Pharmacist), Marlies Ostermann (Consultant), Jennifer Owusu-Afriyie (Research Coordinator), Jacqueline Pan (Data Coordinator), Madeleine Peck (Research Administrator), Gill Radcliffe (Arbane) (Research Manager), Christopher Remmington (Associate PI/Pharmacist), Richelle Santos (Research Nurse), Kyma Morera Vas, (Research Nurse), Francesco Vasques (Consultant), Fionnuala Walton (Research Nurse), Duncan Wyncoll (Consultant)

Hampshire Hospitals NHS Foundation Trust (Royal Hampshire County Hospital and Basingstoke and North Hampshire Hospital), Hampshire England

Irina Grecu (PI), Ana-Maria Arias (Research Nurse), Antony Ashton (Consultant), Regina Askary, (ICU Fellow), Emily Bevan (Physiotherapist), Caroline Wrey Brown (Research Nurse), Maria Alvarez Corral (Research Nurse), Iain Foster (Clinical Trials Assistant), Joseph Goforth (Advance Critical Care Practitioner), Arthur Goldsmith (Consultant), Denise Griffin (Research Nurse), Isi Ikhurionan (Pharmacist), Navneet Kalsi (Consultant), Matthew Lettis (Doctor), Jane Martin (Research Nurse), Catherine McLean (Specialist Pharmacist), Sheila Paine (Clinical Trial Administrator), Lijeesh Pillai (Research Nurse), Jill Salvador (Research Nurse), Mildred Sitonik (Research Nurse), Stephanie Smuk (Research Nurse), Anna Stephenson (Research Nurse), Geoff Watson (Consultant), Lauren Wiltshire (Clinical Trial Administrator)

Heartlands Hospital, Birmingham, England

Daniel Park (PI), Faiz Alam (Pharmacist), Ranjna Basra (Consultant), Neil Crook (Consultant), Neelam Dhansey (Nurse/Associate PI), Eddie Dineen (Senior Physiotherapist), Andrew Gadie (Physician), Jo Gresty (Research Nurse), Angela Holden (Registrar), Karthik Iyer (Physician), Lubna Khan (Pharmacist), Richard Kimberlin (Physiotherapist), Celinamma Mailaykal (Research Nurse), Robert O'Brien (Consultant), Ellie Reeves (Research Nurse), Waqar Riaz (Doctor), Helen Taylor (Data Manager), Gursharan Virdee (Associate PI/Pharmacist), Tisharne Wallace (Physiotherapist), Sarah Milton-White (Consultant), Joyce Yeung (Consultant)

Hull Royal Infirmary, Hull, England

Andrew Gratrix (PI), Felix Allen (Physician), Pete Campbell (Physician), Abigail Clarke (Physician), Abdul Dawood (Physician), Tom Eckersley (Physician), Amy Fleming (Pharmacy), Matthew Hines (Research Nurse), Julian Howes (Physician), Vicky Lowthorpe (Pharmacy), Victoria Martinson (Research Nurse), Alice Poole (Physician), Barbara Ribeiro (Physician), Soumayanil Saha (Physician), Elizabeth Stones (Research Nurse), Karen Winter (Research Nurse)

Ipswich Hospital, Ipswich, England

Robert Lewis (PI), Stephanie Bell (Research Nurse), Paul Carroll (Physician), Emma Galloway (Pharmacist), Mark Garfield (Physician), Emma Galloway (Pharmacist), Richard Howard-Griffin, (Physician), Vicky Jeffrey (Physiotherapist), Paul Mallett (Physician), Eoin McNamee (Clinical Practitioner), Caitlin Palmer (Research Nurse), Sri Nallapareddy (Physician), Vanessa Rivers (Research Nurse), Andrew Taylor (Physician), Kate Turner (Physician)

James Cook University Hospital, Middlesborough, England

Isabel Gonzalez (PI), Andrea Henderson (Physiotherapist), Jeremy Henning (Co-PI), Jessica Coulman (Research Nurse), Hayley Gagiano (Research Nurse), Vicky Hanlon (Pharmacist), Jessica Jones (Research Nurse), Steven Lockwood (Pharmacist), Aileen McAllister (Research Nurse), Emma Pringleton (Pharmacist), Amy Thompson (Research Nurse)

John Radcliffe Hospital, Oxford, England

Ben Attwood (PI), Jon Bailey (Consultant), Graham Barker (Consultant), Jonathan Bedford (Consultant), Sally Beer (Lead Research Nurse), David Clarke (Consultant), Neil Davidson (Research Nurse), Peyton Davis (Consultant), James Day (Consultant), Ben Griffiths (Consultant), Owen Gustafson (Physiotherapist), Raja Jayaram (Consultant), Min Kim (Physiotherapist), Soya Mathew (Research Nurse), Stuart McKechnie (Consultant), Grace Polley (Clinical Research Co-Ordinator/ICU Research Nurse), Bethan Reade (Physiotherapist), Oxford Critical Care Staff (Nurses of all bandings, Allied Healthcare Professionals, Pharmacists, and Occupational Therapists), Samuel Weitzel (Physiotherapist), Jean Wilson (Research Nurse)

King's College Hospital, London, England

Reena Mehta (PI, Pharmacist), Maksym Andreytsiv (Senior Clinical Trials Assistant), Marianette Axalan (Research Nurse), Michael Baker-Kukona (Pharmacist), Aileen Balagot (Research Nurse), Claire Bradley (Clinical Lead Physiotherapist), Anna Broderick (Research Nurse), Milena Chee (Consultant), Vicky Chu (Pharmacist), Emma Clarey (Research Nurse), Eleanor Corcoran (Research Nurse), Maria Theresa Depante (Research Nurse), Asha Farah (Pharmacist), Clare Finney (Research Nurse), Joanna Flanagan (Pharmacy Technician), Joanne Gordon (Pharmacy Technician), Iqra Haq (Pharmacy Coordinator), Philip Hopkins (Consultant), Anthony Leung (Pharmacy Coordinator), Esther Makanju (Pharmacist), Gulam Maxamed (Pharmacy), Maria Nghiem (Senior Clinical Trials Assistant), Harriet Noble (Research Nurse), Gaynor Notcheva (Senior Clinical Trials Technician), Kevin O'Reilly (Co-PI/Research Nurse), Ahmed Osman (Associate PI), Natalie Palmer (Pharmacy Technician), Rohit Saha (Consultant), Leila Samiel (Pharmacist), John Smith (Research Nurse), Caitlin Spooner (Research Nurse), David Tran (Pharmacy Technician), Burt Vergara (Research Nurse), Ekua Wilson (Pharmacy Technician)

Leicester Royal Infirmary, Leicester, England

Simon Scott (PI), Sarah Cooke (Pharmacist Technician), Suzanne Dublin (Clinical Research Practitioner), Rebecca Ealand (Physician), Navneet Ghuman (Clinical Research Practitioner), Jessica Hailstone (Research Nurse), Clair Martin (Associate PI), Taha Masood (Physician), Megha Matthews (Research Nurse), Jess Neville (Clinical Research Practitioner), Ravindra Pochiraju (Physician), Jade Stimson (Research Nurse), Tony Talhat (Physician)

Lincoln County Hospital, Lincoln, England

Adam Wolverson (PI), Rebecca Grinham (PI/Pharmacist), Theja Amarasiri (Anaesthetic Registrar), Heather Baker (Advanced Critical Care Practitioner), Debbie Ball (Physiotherapist/Associate PI), Katie Dorr (Assistant Clinical Research Practitioner), Kelly Hubbard (Research Nurse), Gautam Modak (Anaesthetic Registrar), Teodora Orasanu (Anaesthetic Registrar), Gunjan Phalod (Pharmacy), Louise Potter (Consultant), Ayushka Sinh (Physician), Gemma Smith (Physiotherapist/Associate PI), Catherine Wyatt (Research Assistant)

Manchester Royal Infirmary, Manchester, England

Andrew Bentley (PI), Jonathan Bannard-Smith (Local Lead), Caleb Amporisah-Boama, (Research Nurse), Melanie Barker, (Senior Clinical Research Nurse), Richard Clark (Research Nurse Manager), Carolyn Davies (Pharmacy), Jonathan Finch (Staff Nurse), James Hanison (Physician), Rose Jama (Clinical Research Nurse), Nikki McGill (Physiotherapist), Naeem Meghsee (Physician), Deborah Paripoorani (Research Nurse), Rachael Quayle (Research Nurse), Fionn Quilty (Foundation Doctor),

Betsy Samuel (Senior Clinical Research Nurse), Anila Sukumaron (Senior Clinical Research Nurse), Charlotte Taylor (Senior Clinical Research Nurse), Katharine Wylie (Senior Clinical Trials Co-ordinator), Gregory Yates (Physician)

Medway Maritime Hospital, Gillingham, England
Vipal Chawla (PI), Rebecca Collins (Squires) (Clinical Research Practitioner), Amr Barghout (Physician), Lucy Bates (Physician), Rachel Bowman (Pharmacist), Louise Brassington (Clinical Research Practitioner), Hayley Dolan (Research Nurse), Katrina Kapawen (Research Nurse), Tatenda Kaseke (Physiotherapist), Muhammad Adil Zaka Khan (Physician), Robert Musalagani (Clinical Research Practitioner), Patience Nkala (Clinical Research Practitioner), Linda Ofori (Clinical Research Practitioner), Sandra Sowah (Pharmacist), Anais Sutton (Physiotherapist), Suzanne Williams (Clinical Research Practitioner)

Morriston Hospital, Swansea, Wales
Rowenna Morris-Clarke, (PI), Mark Baker (Research Nurse), Thomas Bull (Physician), David Davies (Physician), Chloe Diamond (Research Nurse), Sean Farley (Research Assistant), Katie Foster (Physiotherapist), Andrew Graham (Physician), Helen Ivatt (Physician), Charlotte Johnston (Physician), Fintan McArdle (Research Assistant), Phillipa Ros, (Physician), Adam Ryan (Physician), Mared Thomas (Physician), Hannah Toghill (Physiotherapist), Paul Watson Jones (Pharmacist), Rebecca Williams (Research Nurse), Nia Wyn Davies (Physician)

Musgrove Park Hospital, Taunton, England
Richard Innes (PI), Richard Bufton (Research Nurse), Richard Burgess (Pharmacist), Rebecca Covey (Research Nurse), Patricia Doble (Research Nurse), Adetutu Lengvenis (Research Assistant Practitioner), Benjamin Plumb (Clinician), Ashly Thomas (Research Nurse)

North Manchester General Hospital, Manchester, England
Andrew Bentley (PI), Shams Abdelraheem (Consultant/Local Lead), Sharon Baxter-Dore (Research Nurse), Andreea Chivu (Pharmacist), Patricia Clohessy (Clinical Trials Assistant), Karen Connolly (Research Practitioner), Claire Fox (Research Nurse), James Hewitt (Junior Doctor), Tracey Hodgkiss (Research Nurse Manager), Bency Laiju (Research Nurse), Mohamed Salah (ICM Trainee), Urbi Sen (Junior Doctor), Kirstie Smith (Research Nurse), Helen T-Michael (Research Nurse), Andy Yang (Pharmacist)

Pinderfields Hospital, Wakefield, England
Brendan Sloan (PI), Ali Alayan (Physician), Oliver Baker (Physician), Alexander Barrett (Physiotherapist), Paige Bayliss-Jones (Physician), Chloe Billingham (Physician), Sarah Buckley (Research Nurse), Robert Burdon (Doctor/Sub Investigator), Gavin Chalmers (Physician), Jonathan Clark (Physiotherapist), Oliver Cohen (Physician), Aaron Corp (Physician), Joanne Cozens (Physician), Abigail Crew (Clinical Trial Administrator), Kate Dalgleish (Physician), Elizabeth Denis (Clinical Trials Assistant), Ayman El-Nayal (Physician), Katherine Emo (Physician), Rowan Farrimond (Physician), Monica Horvath (Physician), Eva Hirst (Physiotherapist), Claire Hutsby, (Pharmacy Technician), Anish Kundu (ICU Doctor), George Humble (Physician), Diana Kluczna (Doctor), Rasmus Kudsen (Doctor), Anna Littlejohns (Doctor), Christopher Macrow (Doctor), Amy Major (Research Nurse), Michal Melgies (Physician), Alexandra Metcalfe (Research Nurse), Rachel Murali-Krishnan (Anaesthetist), Megan Oldbury (Physician), Andrew Peeling (Doctor), Alice Poole (Physician), Megan Pursglove (Clinical Trial Administrator), Emily Rayner (Research Nurse), Alastair Rose (Physician), Jennifer Rothwell (Advanced Critical Care Practitioner), Gitanjali Sharma (Physician), Matthew Smith (Physician), Chloe Start (Physician), Emma-Jayne Stoner (Research Nurse), Daniel Thomas (Doctor), David Tooth (Physician), Benjamin Whitby (Physician), Jessica Wilson (Clinical Trial Administrator), Michael Wright (Advanced Critical Care Practitioner), Nicholas Wroe (Anaesthetist)

Poole Hospital and Royal Bournemouth Hospital, Dorset, England

Nigel Chee, (PI), Leanne Bartlett (Research Nurse), Matthew Bayliss (Data Manager), Rae Bower (Pharmacist), Tim Browning (Physician), Sebastian Croizat (Physician), Yasmin de'Ath, (Research Nurse), Nader Farhead (Physiotherapist), Alison Hogan (Pharmacist), James Keegan (Physician), Emma Langridge (Research Nurse), Nathaniel Merrett (Physiotherapist), Rebecca Miln (Research Nurse), Sally Pitts (Research Nurse), Ken Power (Physician), Sharon Power (Pharmacist), Jenny Rains (Physiotherapist), Javen Ramsami (Data Manager), Henrik Reschreiter (Physician), Sue Roffe (Project Coordinator), Sarah Savage (Project Coordinator), Heather Tiller (Research Nurse)

Queen Alexandra Hospital, Portsmouth, England

David Pogson (PI), Kayode Adeniji (Physician), Alexandra Belcher (Physician), Helen Boxall (Nurse), Helen Claridge (Research Nurse), Richard Clinton (Physician), Jonathan Coates (Consultant), Debbie Cox (Physiotherapist), Gordon Craig (Physician), Zoe Daly (Research Nurse), Dovy Domingo (Nurse), James Doyle (Physician), Shenu Mary George (Research Nurse), Steve Mathieu (Physician), Caroline McCrea (Advanced Critical Care Practitioner), James McNicholas (Physician), Christine Minnis (Pharmacist), Gary Misselbrook (Physician), Angela Nown (Clinical Trials Research Assistant), Helen Peet (Physician), Steve Rose (Research Nurse), David Slessor (Physician), Kelly Tang (Physiotherapist), Nicholas Tarmey (Physician), Philip Young (Physician)

Queen Elizabeth Hospital, Birmingham, England

Dhruv Parekh (PI), Mansoor Bangash (Consultant), Sophia Beddows (Research Nurse), Rebecca Brown, (Research Nurse), Amy Clark (Research Nurse), Amisha Desai (Pharmacist), Caroline Dooley (Research Nurse), Lisa Duncalfe (Research Nurse), Karen Ellis (Research Nurse), Madeleine Harris (Clinical Trial Coordinator), Saffron King (Trial Coordinator), Lisa Moody (Clinical Trial Administrator), Puja Mulholland (Research Nurse), Jaimin Patel (Consultant), Christopher Sheridan (Research Nurse), Hazel Smith (Portfolio Manager), Elaine Spruce (Research Sister), Esther Thomas (Physician), Sumayyah Ul-Rahman (Research Data Manager), Jonathan Weblin (Physiotherapist)

Queen Elizabeth Hospital, Glasgow, Scotland

Christopher Hawthorne (PI), Lynn Abel (Research Nurse), Denise Anderson (Physiotherapist), Rebecca Baird (Research Nurse), Thomas Clift (Physician), Susan Currie (Research Nurse), Carol Dalton (Research Nurse), Steven Henderson (Research Nurse), Fiona Hunter (Research Nurse), Maria Nygren (Clinical Trial Pharmacist), Lynn O'Donohoe (Research Nurse), Izabela Orlikowska (Research Nurse), Jennifer Tait (Clinical Trial Pharmacist)

Queen's Medical Centre, Nottingham, England

Marc Chikhani (PI), Alice Baddeley (Research Nurse), Hannah Blagnys (ICU Registrar), Amy Clark (Research Practitioner), David Ford (Research Nurse), Alexandra Gibbins (Research Nurse), Treesa Joseph (Research Nurse), Sophie Lubbock (Research Nurse), Catherine Mateo (Research Nurse), Alexandra McCoy (Lead Research Nurse), Lisa McLoughlin (Research Nurse), Megan Meredith (Clinical Research Manager), Alistair Morris (Research Practitioner), Geethumol Sasidharan-Nair (Research Nurse), Anthony Ndungu (Research Nurse), Shifa Patel (Research Practitioner), Cecilia Peters (Clinical Research Manager), Andrew Pinder (ICU Consultant), Nicholas Plummer (ICU Consultant), Julia Sampson (Research Nurse), Maria Scott (Lead Pharmacist/Trial Co-Ordinator), Anju Thomas (Research Nurse), Catherine Walton (Research Nurse)

Queen Elizabeth Hospital, London, England

Andy Achilleos (PI), Alistair Bashall (Clinical Fellow), Rebecca Bays (Physiotherapist), Shova Budha (Research Nurse), Shilpa Gurung (Research Nurse), Daniel Harding (Co-Investigator), Nicholas Hoare (Physician), Leanne Howard (Research Nurse), Jak Kalmar (Pharmacist), Dumindu Karangoda

(Physician), Sagira Khatun (Pharmacist), Georgina Luck (Research Nurse), Sinead O'Halloran (Associate Principal Investigator), Estefania Treus (Research Nurse)

Rotherham District Hospital, Rotherham, England

Anil Hormis (PI), Zirlish Afzal (Trial Pharmacist), Damian Burke (Research Nurse), Rachel Dunn (Physiotherapist), Cheryl Graham (Research Nurse), Surinder Kamuta (Trial Pharmacist), Sarah Kimpton (Pharmacist/Associate PI), Jake McCormick (Research Fellow), Susan Oakley (Pharmacy Technician), Jake Warrington (Research Fellow)

Royal Berkshire Hospital, Reading, England

Jodie Smythe (PI), Maryam Amole (Senior Clinical Trials Pharmacist), Jennifer Armistead (Research Nurse), Shauna Bartley (Research Nurse), Parminder Bhuie (Research Nurse), Francesca Compton (Physiotherapist), Georgia Coward (Research Nurse), Sheena Faloon (Research Nurse), Matthew Frise (Consultant), Abigail Goodwin (Trainee Advanced Critical Care Practitioner), Nicola Jacques (Research Sister), Liza Keating (Consultant), Louise Koslicki (Senior Physiotherapist), Giulia Mascia (Research Nurse), Elena Onwualu (Pharmacy Technician), Sabi Rai (Research Nurse), Gabriela Tomkova (Research Nurse), Ada Ukaulor (Research Nurse), Esther Whanya (Pharmacy Technician)

Royal Cornwall Hospital, Truro, England

Mat Holland (PI), Dr Thys DeBeer (PI), David Ashton-Cleary (Consultant), Sarah Bean (Research Nurse), Thys DeBeer (Physician), Karen Burt (Research Nurse), Dave Cain (Physician), Rachel Chapman (Research Nurse), Dave Connor (Physician), Tom Cornell (Research Administrator), Will English (Physician), Michelle Hague (Physiotherapist), Katie Hill (Physiotherapist), Rick Hunt (Physician), Sarah Jarvis (Physician), Liz Kesohole, (Research Nurse), Lorraine Moore (Critical Care Pharmacist), Kate Ralph (Research Administrator), Carol Richards (Research Nurse), Gill Saville (Physician), Barney Scrace (Physician), Michael Spivey (Physician), Will Woodward (Physician)

Royal Devon and Exeter Hospital and North Devon District Hospital, Exeter, England

Thomas Clark (PI), William Gaunt (Consultant/Co-PI–North Devon Site), Katie Adams (Anaesthetics Trainee Doctor), Naomi Adey (Physician), Kizzy Baines (Senior Administrator Officer), Lisa Bashford (Physiotherapist), Abbie Bolus (Research Nurse), Louise Dalton (Senior Advanced Critical Care Practitioner), Samantha Dymond (Specialist Pharmacist), Alexandra Edmonds (Physician), Caterina Ferreira-de-almeida (Research Nurse), Charles Gibson (Physician), Ashley Hanson (Clinical Research Practitioner), Melanie Hutchings (Senior Clinical Trials Administrator), Zaynab Janjua (Physician), Fenella Johnson (Administrator), Samantha Keenan (Lead Research Nurse), Kenneth Ko (Physician), Lynne Van Koutrik (Senior Clinical Research Nurse), Romain Lefebvre (Trainee Advance Critical Care Practitioner Physiotherapist), Andrew Lockwood (Physiotherapist), Nicholas Love (Consultant), Jackie Massey (Research Nurse), Nikita Missula (Physician), Aya Mohamed (Physician), Jennifer Morrison (Research Nurse), Tania Nightingale (Research Nurse), Rebecca Page (Research Nurse), Andrew Pitt (Research Nurse), James Reilly (Physician), Lily Slade (Research Nurse), Mary Stapleton (Consultant Clinical Biochemist), Luke Staveley-Wadham (Physician), Daisy Sykes (Research Nurse), Richard Thomas (Intensive Care Registrar), Cherie Thorn (Nurse), Kevin Thorpe (Senior Administrator Officer), Shirley Todd (Pharmacist), Catherine White (Research Nurse), Elena Williams (Research Support Worker), Ruth Wood (Physiotherapist), Holly Wright (Research Nurse)

Royal Free Hospital, London, England

Mark De Neef (PI), Sharon Augustt (Clinical Trial Coordinator), Torraine Salter (Clinical Lead ICU Physiotherapist), Francesca Gowing (Clinical Trial Coordinator), Charlotte Greenfield (ICU ACP Physiotherapist), Lucy Lloyd (ICU Physiotherapist), Clare Morkane (ICU Consultant), Sarah Pierce (Respiratory Physiotherapist), Sint Sint (Clinical Trial Administrator), Julia Whiteley (Pharmacist)

Royal Hallamshire Hospital and Northern General Hospital, Sheffield Teaching Hospitals NHS Foundation Trust, Sheffield, England

Gary H. Mills (PI), Kris Bauchmuller (Consultant), Kathryn Birchall (Research Nurse), Samuel Dean (Associate PI/Physiotherapist), Helen Ellis (Consultant), Sansha Harris (Research Nurse), Eleanor Meakin Hartill (Research Nurse), Holly Jeffery (Consultant), Jennifer Jennings (Pharmacist), Matthew Needham (Consultant), Max Richardson (Consultant), Andrew Stewart (Consultant), Matthew Wiles (Consultant)

Royal Infirmary of Edinburgh, Edinburgh, Scotland

Thomas Craven (PI), Ruth Alldridge (Physiotherapist), Seth Allen (Physician), Maria Victoria Amamio (Research Nurse), Thomas Anderson (Physician), Lynsey Bagshaw (Pharmacist), Calum Barnetson (Physician), Ruth Begbie (Physician), Sophie Bell (Physician), Sophie Birch (Research Nurse), Michael Blayney (Physician), Kate Briton (Research Nurse), Ruaridh Buchan (Pharmacist), William Calkin (Physician), Lauren Callender (Research Nurse), Keegan Chua (Physician), Sarah Clark (Research Nurse), Kirstin Cleary (Physiotherapist), Rebecca Cowden (Physician), Thomas Cox (Physician), Angela Crawford (Pharmacy Technician), Jessica Crossan (Research Nurse), Coral Darjee (Physician), Simon Davies (Physician), Luke Dornan (Physician), Katherine Doverman (Research Nurse), Stuart Eduardson, (Physician), Rachel Fairlie (Physician), Ross Gillespie (Physician), David Hope (Research Nurse), Alexandra Hunter (Physician), Helen Jordan (Physician), Ancy Joseph (Physician), Stephanie Kelly (Physician), Bara Kubanova (Physician), John Livesey (Physician), Olivia Mansfield (Physician), Lucy Marshall (Research Nurse), Dean McAvoy (Physician), Jonathan Millar (Physician), Steven Morrison (Physician), John Ochiltree (Physician), Emily Ogden (Physician), Harry Putnam (Physician), Tom Quinn (Physician), Krupa Ravi (Physician), Alexander Rollings (Physician), Nabeel Salmin (Physician), Ralph Shackleton (Physician), Manu Shankar-Hari, (Physician), Sunil Sharma (Physician), Scott Simpson (Research Nurse), Jo Singleton (Research Nurse), Iain Slessor (Physician), Zack Slevin (Physician), Duncan Stickle (Physician), Louise Symons (Physician), Sam Talbot (Physician), Ruth Thompson (Administrator), Kate Turnbull (Physician), Maggie Wishart (Senior Research Nurse), Ellen Wisnia (Physician), Rhys Worgan (Physician)

Royal Liverpool University Hospital, Liverpool, England

Ingeborg Welters (PI) Syamlan Ali (API), Ibrahim Almafreji (Research Fellow), Ashwin Balu (Research Fellow), Stephanie Beresford (Research Fellow), Patrick Birkenhead (Research Fellow), Netanya Aarabi Canagarajah (Research Fellow), Josh Colfar (Research Fellow), Alessio Corradini (Research Nurse), Sarah Dyson (Lead Physiotherapist), Jessica Emblem (Research Nurse), Hani Essa (Research Fellow), Sofia Farina (Clinical Fellow), Justyna Gasior (Research Nurse), Edward Gilbert-Kawai (Consultant), Owain Greaves (Physician), Molly Hardwick (Research Fellow), Katherine Hodson (Research Doctor), Brian Johnston (Research Fellow), Caitlin Lythgoe (Research Fellow), Arra Mahiya (Research Nurse), Sylvia Manes (Research Fellow), Maria Lopez Martinez (Research Nurse), Aleem Morenikeji (Research Fellow), Maria Norris (Research Nurse), Jaime Fernandez Roman (Research Nurse), David Shaw (Research Nurse), Sarah Skinner (Pharmacy Technician), Rebecca Tangney (Pharmacist), Zachary Thomas (Research Doctor), Rebecca Varnouse, (Senior Respiratory Physiotherapist), Alicia Waite (Research Fellow), Daniel Watkin (Research Doctor), Victoria Waugh (Research Nurse), Jessica Webber (Research Fellow), Karen Williams (Research Nurse), Yuan Jin-Xi (Research Fellow)

Royal Oldham Hospital, Oldham, England

Redmond Tully (PI), Angiy Vian-Michael (Research Nurse), Abdul Bakhsh (Doctor), Carrole Blessen (Research Nurse), George Caren (Clinical Fellow Junior Doctor), Rebecca Chadwick (Clinical Lead Physiotherapist), Joy Dearden (Research Nurse), Andrew Drummond (Consultant), Emma Dunn (Practice Development Nurse), Faith Elshazly (Doctor), Alex Fitzmaurice-James (Physiotherapist), Sophie Hovenden (Pharmacist), Phillipa Killingworth (Doctor), Whaj Lee (Doctor), William Leeson (Physiotherapist), Helen March (Pharmacist), Michelle Mulcahy, (Research Nurse), Ali Naqvi (Internal

Medical Trainee), Grainne O'Connor (Senior Research Nurse), Richard O'Sullivan (Physician), Arti Rai (Research Assistant), Sarah Winnard (Clinical Trial Administrator)

Royal Preston Hospital, Preston, England

Irfan Chaudry (PI), Linja Akter (Clinical Trial Administrator), Sura Ali (Lead Pharmacist), Sanjog Banstola, (Physician), Lana Bush (Physician), Sacha Connor (Research Nurse), Nathan Fish (Clinical Trial Support Officer), Alex Fonseca (Physician), Lisa Halsall (Advance Critical Care Practitioner), Nayana Jayanna (Physician), Jean Kilroy (Pharmacy Trials Manager), Shondipon Laha (Consultant), Kushal Malleshappa, (Physician), Sean McMullan, (Advance Critical Care Practitioner/Associate PI), Christine Minnis (Pharmacy Manager), Sushruth Raghunath (Physician), Reuben Roy (Physician), Ayman Shaat (Senior Clinical Fellow), Alexandra Williams (Research Nurse)

Royal Stoke University Hospital, Stoke-on-Trent, England

Nehal Patel (PI), Lucy Bailey (Research Practitioner), Liam Botfield (Data Manager), Ciara Brady (Research Administrator), Susan Brammer (Clinical Trials Pharmacist), Bryan Carr (Consultant), Nick Coleman (Consultant), Moses Chikungwa,(Consultant), Michelle Davies (Research Practitioner), Jane Delaney (Clinical Research Assistant), Tom Disney (Consultant), Emma Dunn (Practice Development Nurse), Ingi Eslayed, (Consultant), Alex Fitzmaurice-James (Physiotherapist), Minnie Gellamucho (Senior Research Practitioner), Joanne Hiden, (Senior Clinical Administrator), Lisa Higgins (Advanced Pharmacist), Paul Hollinshead (Pharmacy Technician), Prabhjohyt Kler (Doctor), Stephen Krueper (Consultant), William Leeson (Physiotherapist), Stephen Lord (Consultant), Ramprasad Matsa (Consultant), George Matthew (Locum Consultant), Tony Parlato (Advance Critical Care Practitioner), Divyamol Pathinenchil Joshy (Nursing Assistant), Phoebe Preston (Research Administrator), Fernando Rodriguez-Albarran (Consultant), Deborah Rowley (Advanced Physiotherapist), Timothy Scott (Consultant), Apoorva Singhal (Consultant), Lydia Sterland (Advanced Critical Care Practitioner), Laura Swinscoe (Physiotherapist), Emma Taylor (Advanced Specialist Pharmacist), Christopher Thompson (Consultant), Leah Wiltshaw (Research Practitioner), Sian Wisbey (Research Practitioner), Charlotte York (Research Administrator)

Royal United Hospital, Bath, England

Jerry Nolan (PI), Maxine Brown (Research Nurse), Laura Cini (Pharmacy Assistant), Carrie Demetriou, (Research Nurse), Gabrielle Evans (Research Nurse), Caroline Ficke (Physiotherapist), Kim Hockett, (Clinical Trial Administrator), Lucy Howie (Research Nurse), Jill McDonald-Burn (Pharmacist), Genette Parsons (Research Nurse), Lidia Ramos (Research Nurse), Mark Sheils (Consultant), Tom Simpson (Consultant), Yaya Sodiq (Clinical Trial Administrator), Katy Stevenson (Research Nurse), Roland Wynn-Williams (Pharmacy Technician)

Salford Royal Hospital, Salford, England

Jay Michael Naisbitt (PI), Billie Barratt (Research Nurse), Emma Boxall (Pharmacist), Nathan Butterworth (Research Nurse), Ozerah Choudry (Physician), Reece Jordan Doonan (Research Nurse), Sam Ford (Physician), Tim Fudge (Consultant), Louise Hall (Associate PI), Melanie Handley (Research Nurse), Jack Hodd (Consultant), Jessica Holden (Research Administrator), Daniel Horner (Consultant), Dominic Kay (Research Nurse), Stephanie Lee (Research Nurse), Tracy Marsden (Research Nurse), Danielle McLaughlan (Research Nurse), Dahlia Morris (Research Nurse), Emma Parkin (Advanced Critical Care Practitioner), Jessica Pendlebury, (Research Practitioner), Jane Perez (Research Nurse), Mohamed Salah (Doctor), Daniela Santos (Research Nurse), Rachel Smith (Physiotherapist), Joshua Strange (Doctor), Anila Sukumaran (Research Nurse)

Sandwell General Hospital, Lyndon, England

Deepak Sharma (PI), Satya Anantapatnalkuni, (Doctor), Lubaina Bahar (Consultant), Steven Billingham (Pharmacy), Sumeet Chalal (Trial Pharmacist), Sarala Daram (ICU Doctor), Debbie Devenport (Data Co-

Ordinator), Leila Fares (Pharmacist), Anne Hayes (Research Nurse), Ahmed Hegazy (ICU Consultant), Ellie Higgs (Research Nurse), Lindsay Hough (Pharmacy Technician), Shikshya Khatiwoda, (Doctor), Dean Lavender (Research Nurse), Afelemo Orilade (Doctor), Amy Orme (Research Nurse), Balaji Ramamurthy (Physician), Preksha Sharma (Doctor), Becky Taylor (Research Nurse), Suneesh Thilak (Consultant), Rahul Wakhle (Doctor), Carol Zullo (Senior Research Nurse)

Southmead Hospital, Bristol, England
Matt Thomas (PI), Jessica Annett (Physician), Stella Arthur-Quarm (Physician), Borislava Borislavova (Research Nurse), Ruth Cookson (Research Nurse), Lucy Evans (Pharmacist), Nastassja Findels (Research Nurse), Elizabeth Goff (Research Administrator), Joanna Halls (Research Administrator), Kati Hayes (Research Nurse), Lucy Holmes (Pharmacist), Zunaid Islam (Research Administrator), Georgina Larman (Research Nurse), Ryan Leese (Research Nurse), Heather McLachlan (Research Nurse), Antonia Mentel (Physician), Rebecca Pope (Research Nurse), Ciara Seabrook (Pharmacist), Agnieszka Skorko (Physician), Artur Smalira (Research Nurse), Kerry Smith (Research Nurse), Deanna Stephens (Research Nurse), Jenna Thondee (Pharmacist), Ben Walton (Physician), Ruth Worner (Research Nurse)

Sunderland Royal Hospital, Sunderland, England
Anthony Rostron (PI), David Corbally (Physiotherapist), Sarah Cornell (Research Nurse), Rachel Dimery (Physician), Katherine Doyle (Associate Practitioner), Lyndsey Etherington (Pharmacist), Stephen Laybourn (Pharmacy Technician), Alistair Roy (Consultant), Lindsey Woods (Research Nurse)

Torbay Hospital, Torquay, England
Adam Revill (PI), Kylie Ashby (Administrator), Pauline Aspa (Research Nurse), Kelly Barrett (Research Nurse), Lauren Blunt (Administrator), Sophy Booth (Research Nurse), Adam Carpenter (ICU Consultant), Sean Caunter (Administrator), Olivia Creswell (Physician), Daisy Flanagan (Physician), Emily Flavell (Administrator), Peter Fletcher (Administrator), Angie Foulds (Team Leader), Ashleigh Fynn (Administrator), Isabella Handcock (Physiotherapist), Andrew Harford-Brown, (Pharmacy), Emma Jones (Research Nurse), Chloe Lane (Laboratories), Sally Maddison (Pharmacy), Catherine Marshall (Research Nurse), Pauline Mercer (Research Nurse), Kristy Pearce (Research Nurse), Shraddha Pradhananga (Research Nurse), Bryony Reed (Administrator), Fleur Rogers (Administrator), Claire Stringer (Research Nurse), Ben Whittaker (Physician), Sarah Wright (Pharmacy)

University College London Hospital, London, England
Niall MacCallum (PI), Georgia Bercades (Research Nurse), Clare Black (Physiotherapist), Valeria Boccanera (Research Nurse), David Brealey (Consultant), Laura Gallagher (Research Nurse), Ingrid Hass (Research Nurse), Meenalochani Jegadeesan (Pharmacist), Gladys Martir (Research Nurse), Temi Olusi (Pharmacist), Deborah Smyth (Research Coordinator), Alexandra Zapata (Research Nurse)

University Hospital Coventry, Coventry, England
Bernice Dudkowsky (PI), Thomas Bilyard (Consultant), Carl Hawkins (Research Nurse), Mohammed Mojid Khan (Pharmacy), Katherine Laver (Co-Investigator), Margaret Lindsay (Research Nurse), Anna Ogorek (Clinical Trials Assistant), Maja Ratajczyk (Research Nurse), Saahitya Ganapathi Subrananian (Clinical Trial Assistant Technical Office), Alice Swindell (Research Nurse), Rachel Thompson (Research & Development Team Leader), Aliyah Usman (Pharmacy Clinical Trials Administrative Officer), Geraldine Ward (Research Nurse), Laura Wild (Research Nurse)

University Hospital Lewisham, London, England
Andrew Achilleos (PI), Natalie McLymont, (PI), Rebecca Christie (Physiotherapist), Christine Clarke (Research Nurse), Emily Cox (Physiotherapist), Samantha Dickinson (Pharmacist), Jonathan Drake (Junior Clinical Fellow), Nicholas Beaumont (Consultant), Sagira Khatun (Clinical Trials Pharmacist), Harrison Linford (Physiotherapist), Christine Manalo (Assistant Research Nurse), Mohamed Moubarak

(Senior Clinical Fellow), Alfa Cresia Nilo (Assistant Research Nurse), Selina Rathore (Clinical Trials Pharmacist), Rosie Reece-Anthony (Senior Research Nurse), Kate Setchell (Physiotherapist), Richard Turnor (Clinical Fellow), Maggie Wilson (Lead Physiotherapist), Joe Lam (Vera) Wong (Physiotherapist)

University Hospital Monklands, Airdrie, Scotland
Jim Ruddy (PI), Tracy Baird (Research Nurse Manager), Steven Marshall (Pharmacy), Audrey McAlpine (Research Nurse), Gail McLaughlin (Research Nurse), Dan Silcock (Physician), Emily Yeung, (Physician)

Victoria Hospital, Kirkcaldy, Scotland
Alan Timmins (PI/Pharmacist), Clair Balmain (Clinical Research Assistant), Laura Beveridge (Senior Research Nurse), Tina Coventry (Senior Clinical Research Assistant), Rebecca Cowden (Physician), Caroline Ferguson (Physician), Rhys Greig (Research Nurse), Michael McMahon (Consultant), Anna Morrow (Senior Research Nurse), Kerry Murphy (Physiotherapist)

Warrington Hospital, Warrington, England
Tim Furniss (PI), Lynne Connell (Research Nurse), Suzanne Gurney (Clinical Pharmacist), Charlotte Hancox (Physiotherapist), Rebecca Jones (Physician), Mervin Mahenthran (Anaesthetics Trainee Doctor), Bijoy Mathew (Research Nurse), Joanna Thomas (Lead Physiotherapist), Louise Tyrer-Jones (Pharmacist), Vaclav Vanek (Consultant)

Watford General Hospital, Watford, England
Valerie Page (PI), Ahmed Farouk Abdelaty (PI), Deshmukh, Varun (Physician), Chimenime Ede (Trainee Clinical Trials Assistant), Chiara Ellis (Clinical Trials Assistant), Owen Hardaker (Trainee Research Assistant), Elvira Hoxha (Research Nurse), Lorraine Iveson (Physiotherapist), Ameya Madrekar (Physician), Deepali Oza (Pharmacy), Xiaobei Zhao (Research Nurse)

West Suffolk Hospital, Bury St Edmunds, England
Vijayakumar Gopal (PI), Hebah Afridi (Physician), Sahnia Ali (Pharmacist), James Curtis (Pharmacy Technician), Israa Elfouli (Physician), Victoria Jeffrey (Advance Critical Care Practitioner/Physiotherapist), Rashida Lokhandwala (Physician), Unnathi Manampadi (Clinical Fellow), Parshant Mittal, (Physician), Tim Nye (Physician), Saarah Rana (Physician), Harry Smart (Administrative Assistant), Ruth Tampsett (Research Nurse), Suzahn Wilson (Physiotherapist), Lisa Wood (Research Nurse)

William Harvey Hospital, Ashford, England
Ruth Tighe (PI), Christine Vimalanathan (PI), Altayb Abduelbagi (Physician), Lucy Cooper (Research Nurse), Sam Gibson (Clinical Trials Pharmacy Technician), Rebecca Heatherington (Advance Critical Care Practitioner), Josy Joseph (Advance Critical Care Practitioner), Tanya Plucknett (Physiotherapist), Victoria Priestley (Clinical Research Administrator), Momoh Rabiu (Physician), James Rand (Clinical Research Practitioner), Neil Richardson (Physician), Reanne Solly (Clinical Research Practitioner), Navodhi Somasinghe (Physician), Sarah Stirrup (Research Nurse), Heather Weston (Research Nurse)

Worthing Hospital, Worthing, England
Luke Hodgson (PI), Clinton Corin (Research Nurse), Gina Davies (Research Administrator), Matthew Farrant (Registrar), Jamie Gibson (Co-Investigator), Andy Homes (Research Governance Manager), Kayleigh Jordan (Physiotherapist), Kirsten King (Senior Research Nurse), Samantha Margerison (Research Nurse), Gabrielle McGrath (Clinical Research Fellow), Jake Parnian (Doctor), Thomas Pratt (Consultant), James Richardson (Lead Pharmacist), Sarah Rippin (Facilitator), Emma Sexton (Research Nurse), Rebecca Williams (Doctor), Alice Wright (Physiotherapist)

Wrexham Maelor Hospital, Wrexham, England
Harsha Reddy, (PI), Laura Allen, (Pharmacist), Amy Charnock, (Physiotherapist), Laura Hughes (Research Nurse), Rachel Hughes (Research Nurse), Lisa Jobes (Research Nurse), Laura Shore (Physiotherapist), David Southern (Physician), Heather Thomas (Research Administrator)

Wythenshawe Hospital, Manchester, England
Andrew Bentley (PI), Peter Alexander (Consultant), Barbara Bonvento (Physiotherapist), Patricia Clohessy (Administrator), Hannah Currie (Trial Coordinator), Emmanuel Erhieyovwe (Clinical Research Fellow), Tim Felton (Consultant), Sofia Fiouni (Research Nurse), Sarah Holmes (Research Nurse), Aybaniz Ismayilli (Clinical Research Fellow), Brendan McGrath (Consultant), Naeem Meghjee (Physician), Kirsty Melia (Pharmacy), Rebecca Robey (Physician), Joanne Rothwell (Research Nurse), Katharine Sellers (Trial Coordinator), Luke Ward (Senior Research Nurse)

Yeovil District Hospital, Higher Kingston, England
Sunanda Somisetty (PI), Rupak Kundu (PI), Nigel Beer (Research Nurse), Kate Beesley (Clinical Trial Administrator), Sarah Board (Senior Research Nurse), Rebecca Covey (Research Nurse), Shelley Cullen (Pharmacist), Dave Donaldson (Pharmacy), Jess Foster (Clinical Trial Administrator), Thea Gorman (Physiotherapist), Kirsty Lewis (Research Nurse), Subhro Mitra (Physician), Lucy Pippard (Research Nurse), Agnieska Kubisz Pudelko (Consultant), Apekchhya Pun, (Research Nurse), Aaskaben Shah (Physician), Hayley Stainer (Pharmacy Technician), Lesley Thomas (Pharmacy), Robin Wilcox (Physiotherapist), Emily Williams (Physiotherapist)

York Hospital, York, England
Andrew Chamberlain (PI), Mark Aldous (Clinical Trial Administrator Coordinator), Phil Antill (ICU Consultant), Dominic Burns (Trainee Senior Pharmacy Assistant), Joe Carter (ICU Consultant), Harriet Carter (nee Pearson) (Research Nurse), Callum Childs (Pharmacy Assistant), Zoe Cinquina (Clinical Trials Assistant), Poppy Cottrell-Howe (Pharmacy Clinical Trials Manager), Cheryl Donne (Pharmacy Technician), Rob Ferguson (ICU Consultant), Arran Fletcher (Senior Pharmacy Assistant), Daniel Greenwood (Associate PI/Resident Doctor), Felix Harmer (Research Nurse), Tanya Hartley (Trainee Senior Pharmacy Assistant), Angela Hewitt (Pharmacy Assistant), Jo Ingham (Research Nurse), Sharon Jardine (Senior Pharmacy Assistant), Vicki Jones (Clinical Trial Assistant), Rebecca Lahney (Trainee Clinical Trials Pharmacy Assistant), Kate Laurence (Research Allied Health Practitioner), Clare Panaser (Research Midwife), Elizabeth Percy (Clinical Trials Assistant), George Priestley (ICU Consultant), Jonathan Redman (ICU Consultant), Barbara Ribeiro (Associate PI/Resident Doctor), Caroline Savage (Senior Pharmacy Assistant), Zoe Scott (Research Nurse), Rachel Spooner (Pharmacy Technician), Sue Urwin (ICU Consultant), David Yates (ICU Consultant), Elaine Zocatelli (Research Nurse)

SUPPLEMENTARY METHODS

S1. Eligibility criteria

Eligibility criteria were the same for each factor in the factorial design and were designed to allow enrolment of a broad and generalizable population of critically ill patients who may benefit from the therapeutic intervention, while excluding patients who may be more likely to experience an adverse reaction. Eligibility criteria are presented below, as per the Trial Protocol[1] (2024 12 02 MARCH Protocol Final V4.0).

Inclusion criteria

1. Aged ≥16 years
2. An acute and potentially reversible cause of acute respiratory failure as determined by the treating physician
3. Receiving invasive mechanical ventilation via endotracheal tube or tracheostomy
4. Anticipated to remain on invasive mechanical ventilation for at least 48 hours
5. Presence of secretions that are difficult to clear with usual airway clearance management (as assessed by the treating clinical team)

Exclusion criteria

1. Pre-existing chronic respiratory condition receiving routine use of any mucoactive
2. Mucoactive treatment started more than 24 hours prior to trial enrolment
3. Known adverse reaction to either study mucoactive
4. Treatment withdrawal expected within 24 hours
5. Known pregnancy
6. Previous enrolment in the MARCH trial
7. Declined consent
8. The treating physician believes that participation in the trial would not be in the best interests of the patient

S2. Usual care and mucoactive interventions

All participants received usual care; all aspects of intensive care management were delivered according to standard critical care guidelines. Usual care included usual airway clearance management approaches[2] - suctioning, heated humidification (active or passive), respiratory physiotherapy airway clearance, and use of isotonic saline at individual clinician discretion.

Respiratory physiotherapy airway clearance management was not protocolized but delivered at the discretion of treating physiotherapists based on assessment of the individual clinical need of patients[2]. The frequency, duration, and content of treatment sessions therefore varied among patients. However, typical airway clearance management is characterized by tailored treatment according to the specific patient presentation using a range of available techniques and evaluated using both subjective and objective outcome measures[2]. As is the case in usual clinical practice, individual treating physiotherapists were able to schedule their treatment sessions in combination with the delivery of the study mucoactives to optimize patient management.

Carbocisteine is a mucoregulator, where the mechanism of action is to redress the balance between the glycoproteins that regulate mucus viscosity, thereby restoring typical viscoeleastic properties. Conversely, hypertonic saline is an expectorant, where the mechanism of action is to thin mucus by increasing mucosal water volume which facilitates sputum expectoration via maintenance of the periciliary layer and decreasing mucus adhesiveness, in addition to stimulating cough reflexes.

S3. Criteria for discontinuing or modifying allocated interventions

Study mucoactives (whether carbocisteine, or hypertonic saline, or both) were continued until the first of the following:

1. 28 days elapse since randomization
2. First successful unassisted breathing
3. Study mucoactive-related serious adverse event
4. Discharge from the intensive care unit
5. Death or discontinuation of active medical treatment
6. Request from Legal Representative or patient to withdraw from the trial
7. Decision from the attending intensive care unit physician that the study mucoactive should be discontinued on safety grounds

S4. Clarifications and working examples for the primary outcome

The primary outcome was the duration of mechanical ventilation (hours) defined as the time from randomization until first successful unassisted breathing (maintaining unassisted breathing at 48 hours) or death.[11] Specific clarifications are as follows:

i) Unassisted breathing defined as no inspiratory support or extracorporeal lung support

ii) Success defined as maintaining unassisted breathing at 48 hours

iii) Duration included time receiving extracorporeal lung support, invasive mechanical ventilation and non-invasive ventilation delivering volume or pressure support ventilation

iv) Duration excluded time receiving high-flow oxygen therapy and continuous positive airway pressure

v) Patients with a tracheostomy or endotracheal tube in situ could still achieve successful unassisted breathing

vi) Follow-up to 60 days from randomization

vii) Measure time by calculating date/time of start and stop; do not include the 48-hr success period (NB: unsuccessful periods of unassisted breathing (i.e. <48 hours) are considered part of the duration of mechanical ventilation until success (the 48-hour threshold) is reached)

viii) Present data for survivors and non-survivors

ix) Case Report Form items: Date/time of randomization; date/time of first successful unassisted breathing; date/time of death (NB: the 48-hour success period is deducted during analysis)

Examples to reflect data capture and calculation of the duration of mechanical ventilation:

Example 1

Patient AB randomized, 1000 04/03/2024

- Extubated onto regular oxygen therapy, 2200 05/03/2024

- Period of unassisted breathing commences, patient remains on regular oxygen therapy for 48 hours
- Achieved successful unassisted breathing (primary outcome), 2200 07/03/2024
- Duration of mechanical ventilation: (2200 07/03/2024 - 1000 04/03/2024) - 48 hours

  = (84 hours) – 48 hours

  = 36 hours

Example 2

Patient CD randomized, 1400 04/12/2023

- Extubated on to CPAP, 1530 15/12/2023
- Period of unassisted breathing commences, patient remains on CPAP for 48 hours
- Achieves successful unassisted breathing (primary outcome), 1530 17/12/2023
- Duration of mechanical ventilation: (1530 17/12/2023 - 1400 04/12/2023) - 48 hours

  = (313.5 hours) – 48 hours

  = 265.5 hours

Example 3

Patient EF randomized, 1000 02/01/2024

- Extubated onto NIV, 10/01/2024
- Transition from NIV to high-flow oxygen, 1700 16/01/2024
- Period of unassisted breathing commences, patient remains on high flow oxygen for 48 hours
- Achieved successful unassisted breathing (primary outcome), 1700 18/01/2024
- Duration of mechanical ventilation: (1700 18/01/2024 - 1000 02/01/2024) – 48 hours

  = (391 hours) – 48 hours

  = 343 hours

Example 4

Patient GH randomized, 1100 08/03/2024

- Weaned to CPAP, 1030 10/04/2024
- Period of unassisted breathing commences, patient remains on CPAP for 12 hours and resumes assisted mode of ventilation e.g. Pressure Support, Pressure Control
- Weaned to CPAP, 1600 14/04/2024, patient remains on CPAP for 48 hours
- Achieved successful unassisted breathing (primary outcome), 1600 16/04/2024
- Duration of mechanical ventilation: (1600 16/04/2024 - 1100 08/03/2024) – 48 hours

  = (941 hours) – 48 hours

  = 893 hours

S5. Complier Average Causal Effect analysis

To estimate the causal effect of carbocisteine and hypertonic saline among patients who complied with their randomised allocation, a post hoc complier average causal effect analysis was conducted using a two-stage residual inclusion approach. Randomised allocation was used as an instrumental variable for receipt of the intervention (carbocisteine or hypertonic saline). In the first stage, receipt of intervention was modelled as a function of randomised allocation. In the second stage, a Cox proportional hazards model including treatment receipt and the first-stage residuals was fitted, adjusting for age and APACHE II score, with shared gamma frailty to account for clustering by site.

SUPPLEMENTARY FIGURES

S1. CONSORT flow diagram by treatment group

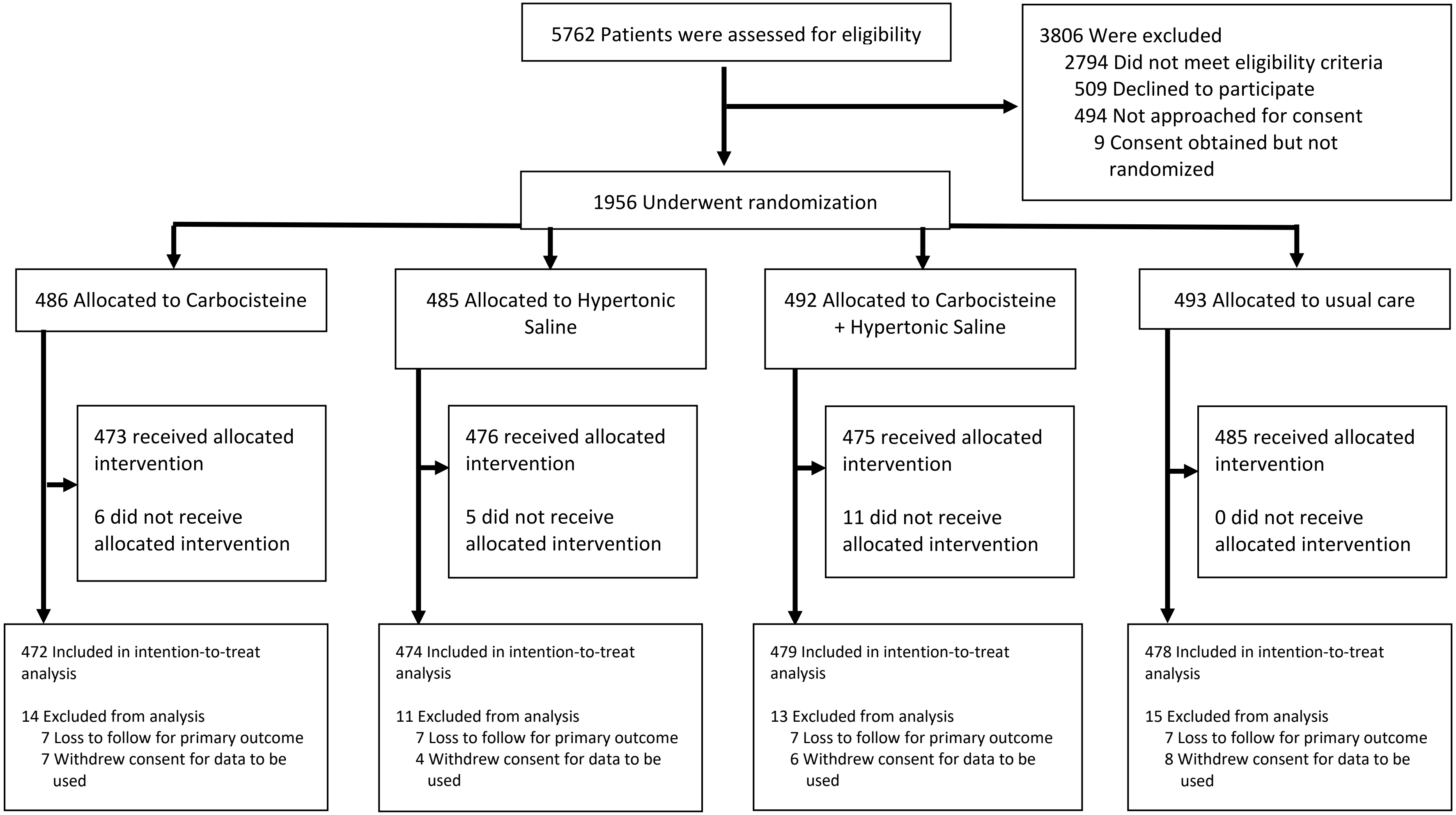

Figure S1. CONSORT flow diagram by treatment group

S2. Kaplan-Meier curves: Primary outcome (Intention-to-treat population)

A

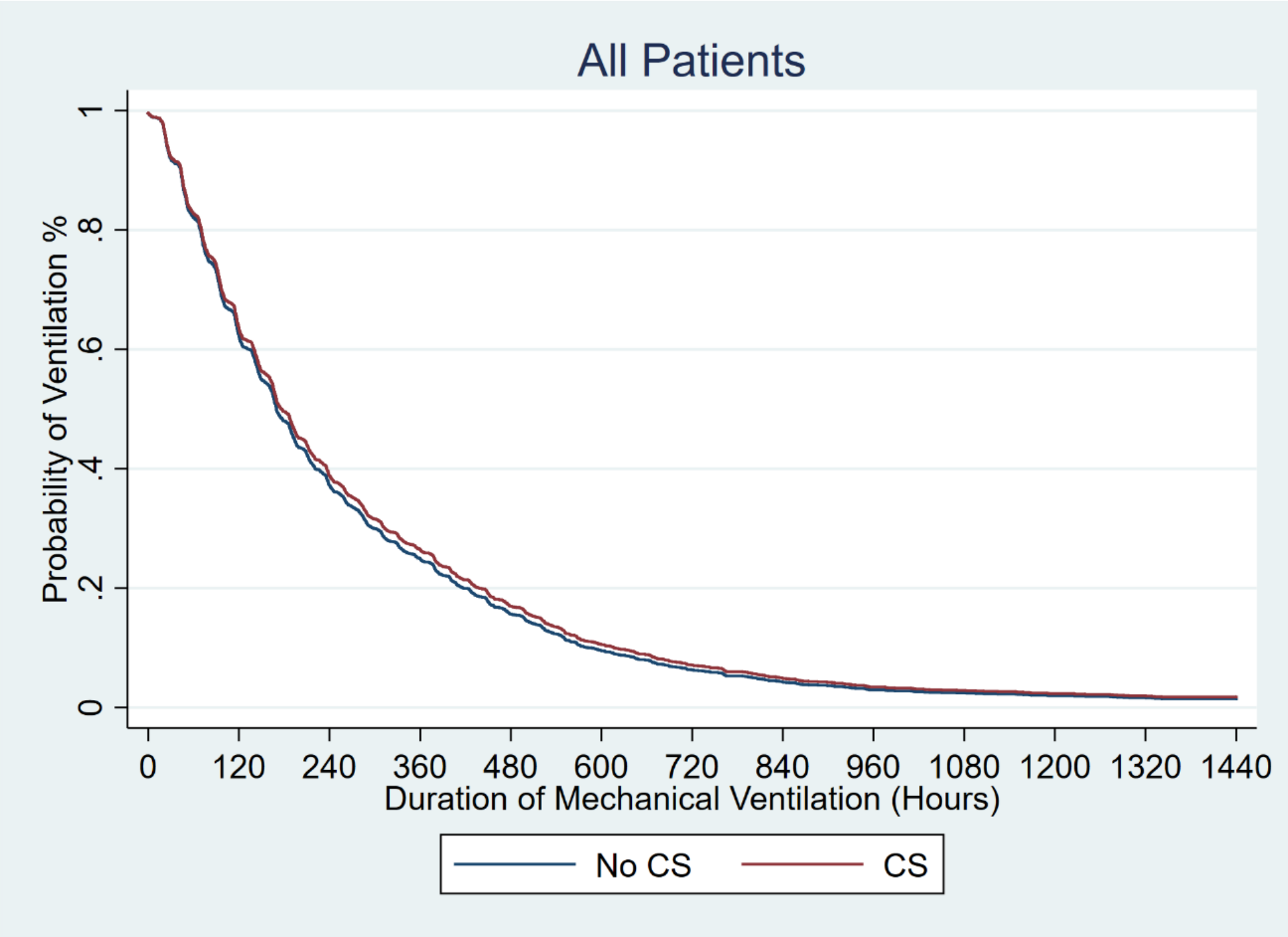

B

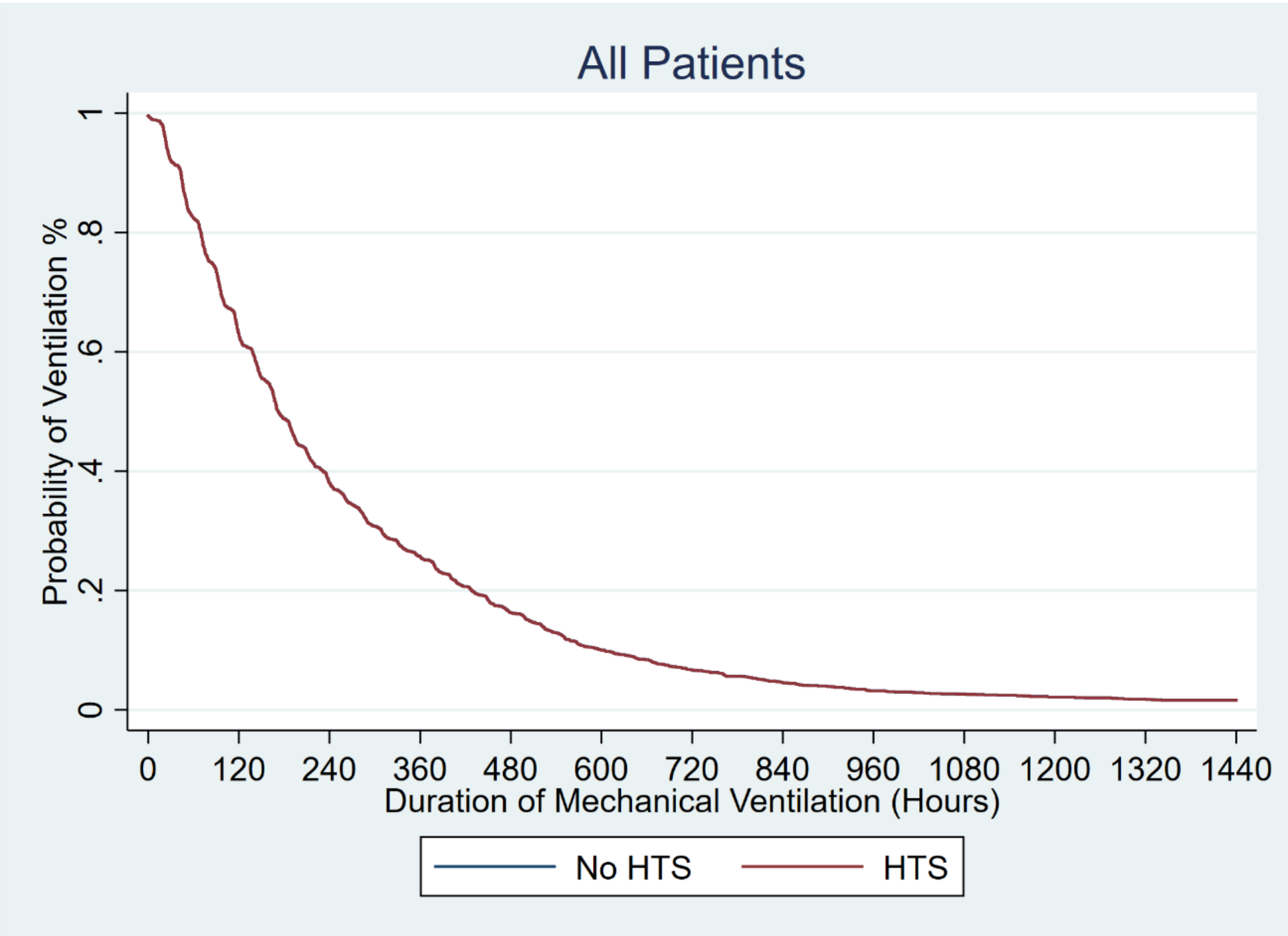

Figure S2. Kaplan-Meier curves for duration of mechanical ventilation (intention-to-treat population)

A. Carbocisteine (CS) versus no carbocisteine comparison. B. Hypertonic saline (HTS) versus no hypertonic saline comparison.

S3. Kaplan-Meier curves: Primary outcome (Survivors)

A

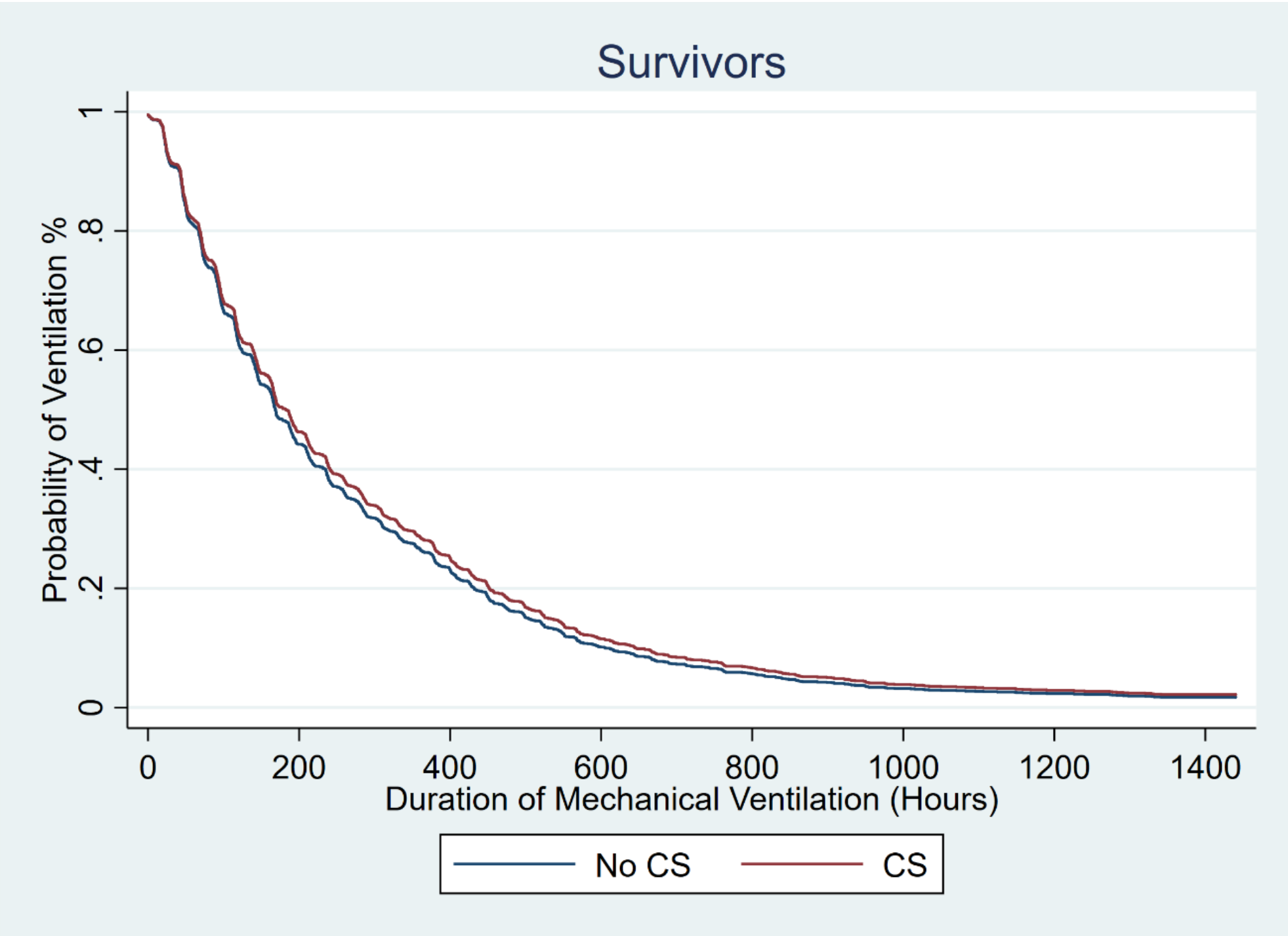

B

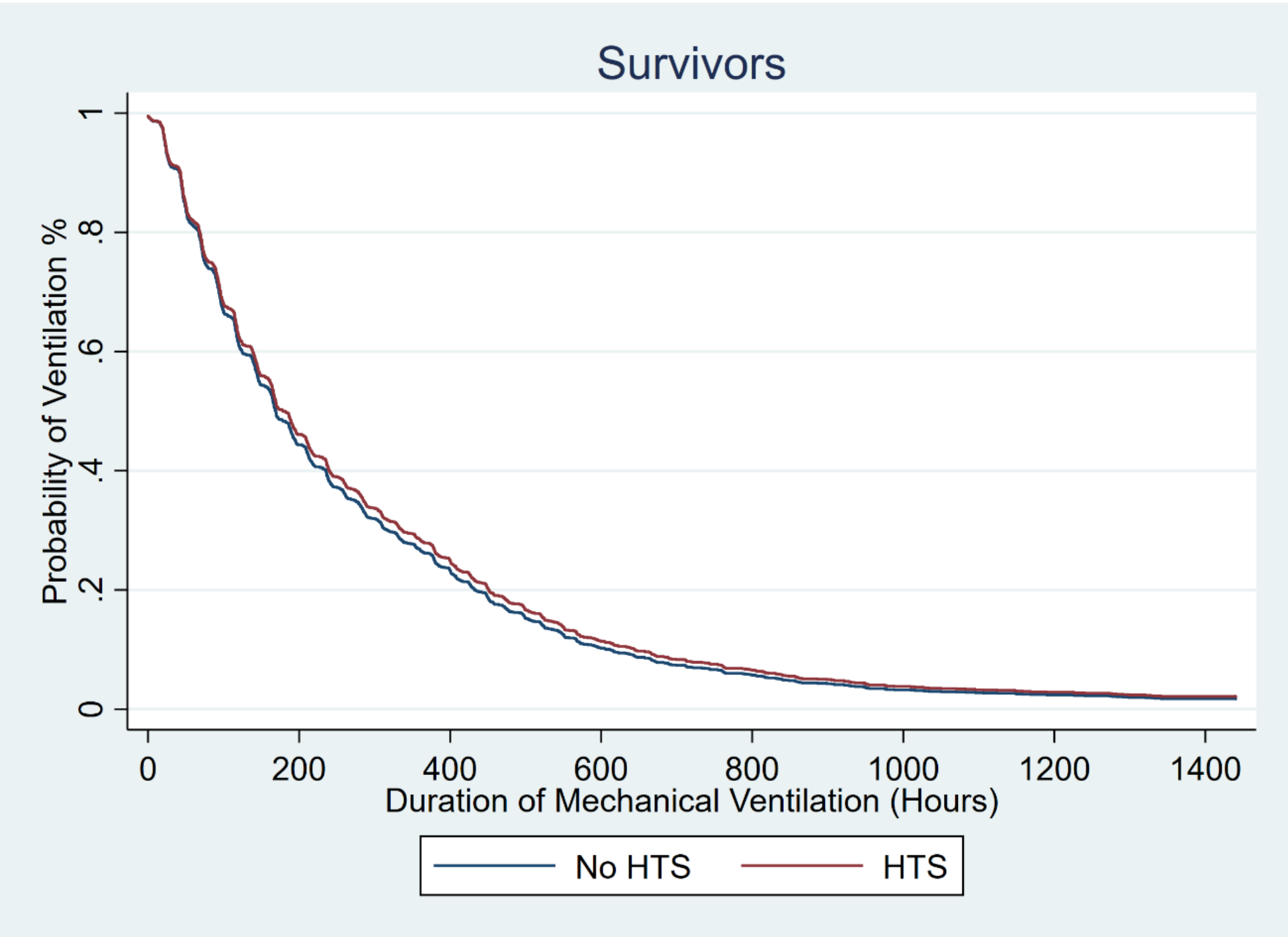

Figure S3. Kaplan-Meier curves for duration of mechanical ventilation (survivors)

A. Carbocisteine (CS) versus no carbocisteine comparison. B. Hypertonic saline (HTS) versus no hypertonic saline comparison.

S4. Kaplan-Meier curves: Primary outcome (Non-survivors)

A

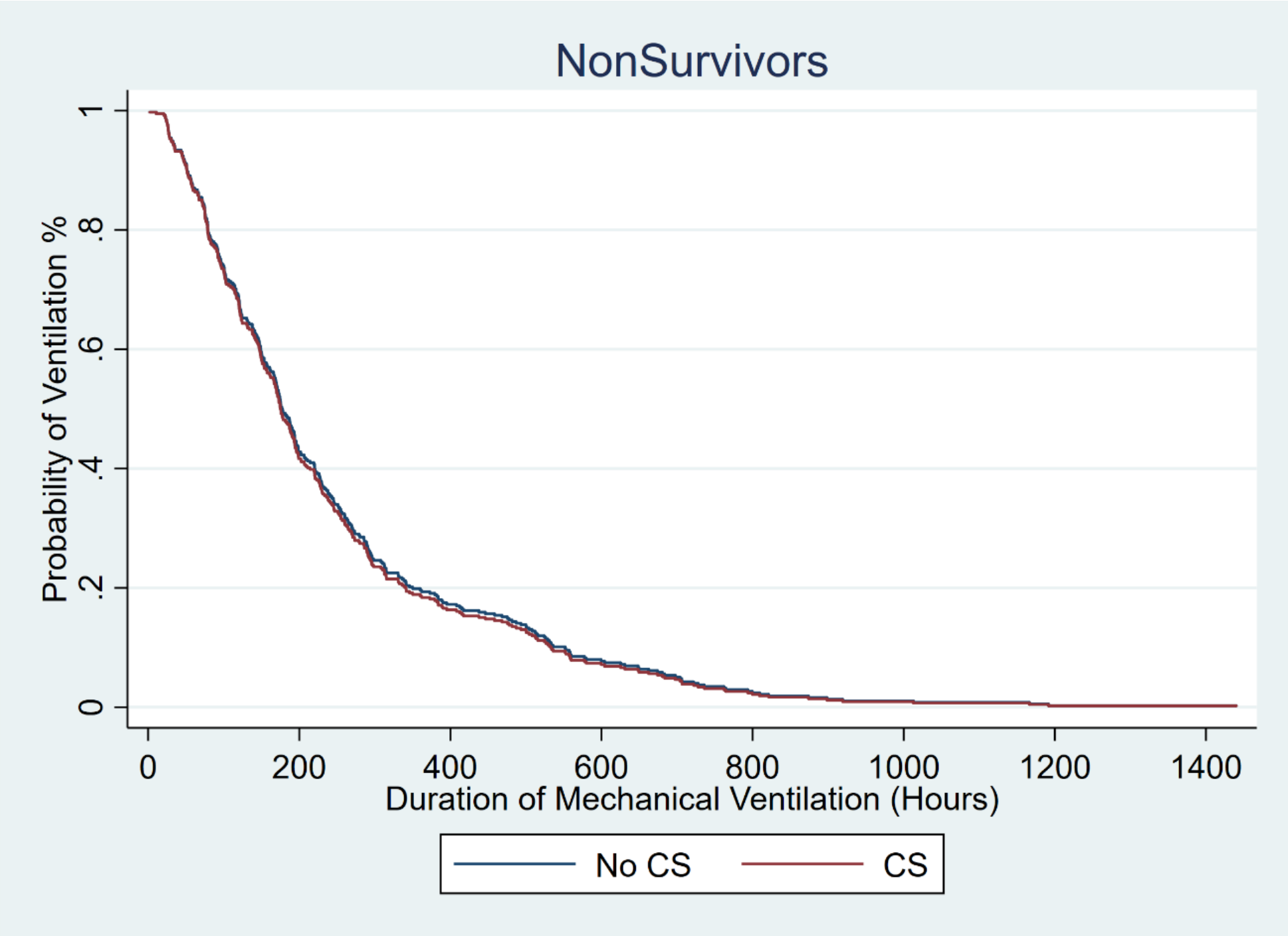

B

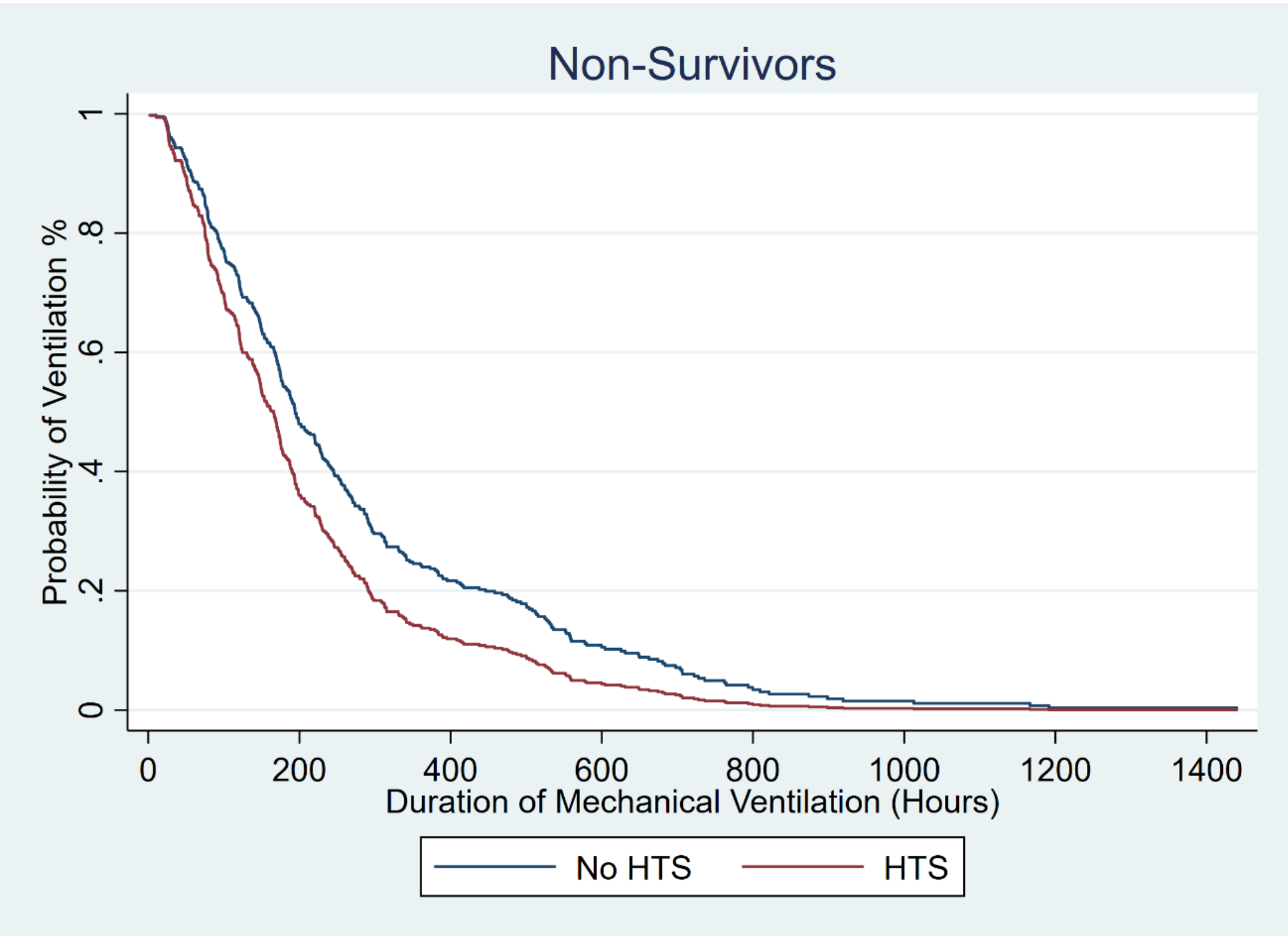

Figure S4b. Kaplan-Meier curves for duration of mechanical ventilation (Non-survivors)

A. Carbocisteine (CS) versus no carbocisteine comparison. B. Hypertonic saline (HTS) versus no hypertonic saline comparison.

SUPPLEMENTARY TABLES

S1. Site recruitment summary

Table S1. Site recruitment summary

Site name	Number of participants recruited
Bristol Royal Infirmary, Bristol, England	160
Royal Stoke University Hospital, Stoke-on-Trent, England	151
Guy’s and St Thomas’ NHS Foundation Trust, London, England	115
Belfast City Hospital and Royal Victoria Hospital, Belfast, Northern Ireland	91
Freeman Hospital and Royal Victoria Infirmary Hospital, Newcastle Upon Tyne, England	66
King's College Hospital, London, England	66
Aintree Hospital, Liverpool, England	55
Addenbrookes Hospital, Cambridge, England	45
Royal Oldham Hospital, Oldham, England	44
Sunderland Royal Hospital, Sunderland, England	43
Hull Royal Infirmary, Hull, England	40
Southmead Hospital, Bristol, England	39
Royal Devon and Exeter Hospital and North Devon District Hospital, Exeter, England	39
John Radcliffe Hospital, Oxford, England	38
Royal Liverpool University Hospital, Liverpool, England	37
Poole Hospital, Dorset, England	37
Golden Jubilee University National Hospital, Clydebank, Scotland	36
Hampshire Hospitals NHS Foundation Trust (Royal	34
Pinderfields Hospital, Wakefield, England	34
Queen Alexandra Hospital, Portsmouth, England	33
Royal Berkshire Hospital, Reading, England	32
Glasgow Royal Infirmary, Glasgow, Scotland	30
Derriford Hospital, Plymouth, England	30
University Hospital Lewisham, London, England	29
Watford General Hospital, Watford, England	28
James Cook University Hospital, Middlesborough, England	28
Royal Bournemouth Hospital, Dorset, England	27
Warrington Hospital, Warrington, England	27
Royal Infirmary Edinburgh, Edinburgh, Scotland	26
York Hospital, York, England	25
Aberdeen Royal Infirmary, Aberdeen, Scotland	25
Queen’s Medical Centre, Nottingham, England	25
Manchester Royal Infirmary, Manchester, England	24

Royal Cornwall Hospital, Truro, England	21
University College London Hospital, London, England	20
Medway Maritime Hospital, Gillingham, England	19
Royal Preston Hospital, Preston, England	17
Lincoln County Hospital, Lincoln, England	17
West Suffolk Hospital, Bury St Edmunds, England	17
Heartlands Hospital, Birmingham, England	16
Royal Free Hospital, London, England	16
Gloucestershire Royal Hospital, Gloucester, England	15
Glan Clywd Hospital, Rhyl, Wales	15
Musgrove Park Hospital, Taunton, England	15
Royal Hallamshire Hospital and Northern General Hospital, Sheffield, England	14
Royal United Hospital, Bath, England	14
Queen Elizabeth Hospital, London, England	14
Morriston Hospital, Swansea, Wales	13
Altnagelvin Hospital, Londonderry, Northern Ireland	12
Chesterfield Royal Hospital, Chesterfield, England	12
William Harvey Hospital, Ashford, England	12
Victoria Hospital, Kirkcaldy, Scotland	11
Grange University Hospital, Cwmbrân, Wales	11
Queen Elizabeth Hospital, Glasgow, Scotland	11
Wythenshawe Hospital, Manchester, England	10
Rotherham District Hospital, Rotherham, England	8
Leicester Royal Infirmary, Leicester, England	8
Yeovil District Hospital, Higher Kingston, England	8
Barnsley Hospital, Barnsley, England	7
University Hospital Coventry, Coventry, England	7
Bedford Hospital, Bedford, England	6
Ipswich Hospital, Ipswich, England	5
Queen Elizabeth Hospital, Birmingham, England	5
University Hospital Monklands, Airdrie, Scotland	5
Torbay Hospital, Torquay, England	5
Salford Royal Hospital, Salford, England	4
North Manchester General Hospital, Manchester, England	4
Sandwell General Hospital, Lyndon, England	4
Wrexham Maelor Hospital, Wrexham, England	2
Antrim Area Hospital, Antrim, Northern Ireland	1
Worthing Hospital, Worthing, England	1
**TOTAL**	**1956**

Note: Final absolute numbers of patients reported does not account for duration of site time open to recruitment

S2. Diversity

Table S2. Representativeness of Study Participants and Sites

**Category**	**Comment**
Disease, problem, or condition under investigation	Acute respiratory failure (critical illness)
Special considerations related to	
Sex and gender	The national Case Mix Programme audit of critical care (2022-23)[3] reports that 58.3% of patient admissions to UK critical care units were male
Age	The national Case Mix Programme audit of critical care admissions (2022-23)[3] reports that the average age of patients was 60.6±16.8 years
Race or ethnic group	The national Case Mix Programme audit of critical care admissions (2022-23)[3] reports that the race or ethnic grouping of patients was as follows:<br><br>White: 78.7%<br>Mixed/multiple ethnic groups: 0.8%<br>Asian/Asian British: 4.9%<br>Black/African/Caribbean/Black British: 2.9%<br>All others: 2.4%<br>Not stated: 10.3%
Geography	The 71 sites that participated in the MARCH trial proportionately reflected all four United Kingdom nations (n=57 England, n=7 Scotland, n=4 Wales, n=3 Northern Ireland). Using national classification systems and definitions[4-7], the 71 sites represent the following geographical categories:<br><br>Urban: n=53<br>Coastal: n=12<br>Rural: n=6
Other considerations	The national Case Mix Programme audit of critical care (2022-23)[3] reports that the range of socioeconomic status of patient admissions to UK critical care units, measured using the Index of Multiple Deprivation was as follows:<br><br>1 (least deprived): 17.1%<br>2: 18.6%<br>3: 20.1%<br>4: 21.5%<br>5 (most deprived): 22.7%<br><br>Using corresponding classification systems, the range of socioeconomic status of sites in the MARCH trial is:<br><br>1 (least deprived): 21.1%<br>2: 23.9%<br>3: 21.1%<br>4: 19.7%<br>5 (most deprived): 14.1%
Overall representativeness of this trial	Sex<br>- The % male participants for each of the trial groups is around 70% which is slightly higher compared to the national average<br><br>Age:

	- The average age of participants for each of the trial groups is around 57 years which is consistent with the national average<br><br>Race or ethnic group:<br>- Across the trial groups, the proportion of patients from a White race or ethnic group is slightly higher (around 86%) than national audit data. Other race or ethnic groups are broadly consistent accounting for differences in data collection/reporting of options (race or ethnic group options in the trial were derived from UK census options and collected from reported information in medical notes)<br><br>Geography<br>- MARCH trial sites reflect a good diversity of geographical locations across the UK and in terms of nature of setting<br><br>Socioeconomic status<br>- MARCH sites are in areas reflecting a good mix of socioeconomic status. The profile of ranges differs somewhat from the national average as we have looked at socioeconomic status at the level of a site, rather than at an individual participant level (as the national audit does). It is likely that one site serves a region encompassing a more diverse mix of participants spanning socioeconomic status

S3. Secondary outcomes

Table S3. Detail of secondary outcomes

Outcome	Measurement tool, definition, method
**In hospital**	
Extubation	Time from randomization to first successful extubation (success defined as remaining free from endotracheal or tracheostomy tubes at 48 hours)<br>Censored at 60 days
Re-intubation	Event of reintubation of endotracheal tube after a planned extubation Excludes temporary reinsertion of endotracheal tube for procedures only; Censored at 60 days
Respiratory physiotherapy input	Occurrence and frequency of airway clearance sessions<br>Censored at Day 28 (or the primary outcome is reached), or ICU discharge, or death, whichever comes first*
Antibiotic usage	Dose of individual agents<br>Censored at Day 28 (or the primary outcome is reached), or ICU discharge, or death, whichever comes first*
Duration of ICU and hospital stay	Time from randomization until patient first leaves the relevant facility or dies<br>Censored at 6 months
All-cause mortality	Confirmation and cause of death
Safety	i) Clinically important upper gastrointestinal (GI) bleeding due to peptic ulceration confirmed on upper GI endoscopy<br>- Defined as overt bleeding on upper GI endoscopy, developing as a complication in the ICU and accompanied by 1 or more of the following features within 24 hours:<br>1) Spontaneous drop of systolic, mean arterial pressure or diastolic blood pressure of 20 mmHg or more<br>2) Start of vasopressor or a 20% increase in vasopressor dose<br>3) Decrease in hemoglobin of at least 2 g/dl<br>4) Transfusion of 2 units of packed RBC or more[8]<br><br>ii) Bronchoconstriction requiring nebulized bronchodilators<br>- During or up to 30 minutes following nebulisation[9]<br><br>iii) Ventilator or circuit dysfunction with respiratory deterioration<br>- This may include hypoventilation, hypoxemia, or other signs of respiratory deterioration temporally associated with ventilator or ventilator circuit dysfunction[10]<br><br>iv) Hypoxemia during nebulization<br>- A drop in SpO2 to below 90% during or up to 30 minutes following nebulization requiring an increase in FiO_2 [9]<br><br>Censored at Day 28 (or the primary outcome is reached), or ICU discharge, or death, whichever comes first*
Hospital resource use	Number of days at Level of Care 0/1/2/3

	Censored at 6 months
**Time of consent to continue**	
Health-related quality of life	EQ-5D-5L<br>*EuroQol-5 Dimension-5 Level (EQ-5D-5L)*<br>- A generic preference-based measure of health, which provides a description of health using five dimensions (mobility, self-care, usual activities, pain and discomfort, and anxiety and depression) each with five levels of severity ranging from no to extreme problems. Responses can be converted to an overall utility score and used for the calculation of quality adjusted life years. Respondents are also asked to place their health on a visual analogue scale (VAS) where 0 represents the worst imaginable health state and 100 the best imaginable health state.
**60 days**	
Health-related quality of life	EQ-5D-5L
All-cause mortality	Confirmation and cause of death
**6 months**	
Health-related quality of life	EQ-5D-5L
All-cause mortality	Confirmation and cause of death
Health service use since hospital discharge	Categories: care at hospital, emergency, GP surgery, health clinic, or other community setting, health care at home, medication

*Where unassisted breathing began on Day 27 or Day 28, data were recorded up to Day 29 or Day 30 respectively. ICU = intensive care unit. GP = General Practitioner (primary care physician). Hospital resource/service use and health-related quality of life data (across hospital, Day 60, and 6-month timepoints) will be reported separately in the pre-planned health economic analysis.

S4. Additional characteristic data of participants at baseline

Table S4. Additional characteristic data of participants at baseline

Characteristic*	Carbocisteine (N=965)	No carbocisteine (N=966)	Hypertonic saline (N=967)	No hypertonic saline (N=964)
**Functional Comorbidity Index categories**				
- **Angina**	34 (3.5)	25 (2.6)	33 (3.4)	26 (2.7)
- **Anxiety or panic disorders**	136 (14.1)	129 (13.4)	145 (15.0)	120 (12.5)
- **Arthritis *(RA or OA)***	70 (7.3)	79 (8.2)	64 (6.6)	85 (8.8)
- **Asthma**	101 (10.5)	130 (13.5)	115 (11.9)	116 (12.0)
- **COPD, ARDS, or emphysema**	107 (11.1)	112 (11.6)	123 (12.7)	96 (10.0)
- **CHF *(or heart disease)***	103 (10.1)	83 (8.6)	99 (10.2)	87 (9.0)
- **Degenerative disc disease[1]**	50 (5.2)	48 (5.0)	52 (5.4)	46 (4.8)
- **Depression**	186 (19.3)	180 (18.6)	185 (19.1)	181 (18.8)
- **Diabetes (Types I and II)**	197 (20.4)	193 (20.0)	185 (19.1)	205 (21.3)
- **Hearing impairments[2]**	15 (1.6)	17 (1.8)	13 (1.3)	19 (2.0)
- **Heart Attack *(MI)***	75 (7.8)	63 (6.5)	67 (6.9)	71 (7.4)
- **Neurological disease[3]**	28 (2.9)	24 (2.5)	25 (2.6)	27 (2.8)
- **Obesity and/or BMI > 30**	260 (26.9)	233 (24.1)	258 (26.7)	235 (24.4)
- **Osteoporosis**	19 (2.0)	18 (1.9)	21 (2.2)	16 (1.7)
- **Peripheral Vascular Disease**	24 (2.5)	35 (3.6)	27 (2.8)	32 (3.3)
- **Stroke or TIA**	53 (5.5)	52 (5.4)	55 (5.7)	50 (5.2)
- **Upper GI disease[4]**	88 (9.1)	92 (9.5)	78 (8.1)	102 (10.6)
- **Visual Impairment[5]**	32 (3.3)	38 (3.9)	33 (3.4)	37 (3.8)
**ICU admission diagnostic category**				
- **Orthopedics/trauma**	72 (7.5)	76 (7.9)	75 (7.8)	73 (7.6)
- **Surgical/medical procedures**	59 (6.1)	60 (6.2)	55 (5.7)	64 (6.6)
- **Gastrointestinal**	55 (5.7)	52 (5.4)	56 (5.8)	51 (5.3)
- **Infection**	50 (5.2)	56 (5.8)	54 (5.6)	52 (5.4)
- **Toxicology/poisoning**	40 (4.2)	37 (3.8)	44 (4.6)	33 (3.4)
- **Hepatobiliary**	33 (3.4)	37 (3.8)	36 (3.7)	34 (3.5)
- **Renal and urinary**	19 (2.0)	8 (0.8)	16 (1.7)	11 (1.1)
- **Endocrine**	13 (1.4)	13 (1.4)	14 (1.5)	12 (1.2)
- **Injury**	9 (0.9)	6 (0.6)	10 (1.0)	5 (0.5)
- **Hematology**	3 (0.3)	5 (0.5)	5 (0.5)	3 (0.3)
- **Other**	9 (0.9)	11 (1.1)	11 (1.1)	9 (0.9)
**Chronic respiratory condition**				
- **COPD**	90 (45.5)	90 (42.5)	98 (45.2)	82 (42.5)
- **Asthma**	77 (38.9)	93 (43.9)	90 (41.5)	80 (41.5)

- **Obstructive sleep apnea**	7 (3.5)	8 (3.8)	7 (3.2)	8 (4.2)
- **Sarcoidosis**	4 (2.0)	1 (0.5)	1 (0.5)	4 (2.1)
- **Interstitial Lung Disease**	2 (1.0)	4 (1.9)	3 (1.4)	3 (1.6)
- **Lung Cancer**	2 (1.0)	6 (2.8)	5 (2.3)	3 (1.6)
- **Bronchiectasis**	2 (1.0)	1 (0.5)	2 (0.9)	1 (0.5)
- **Cystic Fibrosis**	0 (0.0)	0 (0.0)	0 (0.0)	0 (0.0)
- **Other**	14 (7.0)	9 (4.3)	11 (5.1)	12 (6.2)
**Neurological diagnosis**				
- **Stroke**	64 (26.7)	82 (32.0)	68 (28.1)	78 (30.7)
- **Head Injury**	49 (20.4)	54 (21.1)	50 (20.7)	53 (20.9)
- **Epilepsy**	37 (15.4)	30 (11.7)	30 (12.4)	37 (14.6)
- **Guillain-Barre Syndrome**	18 (7.5)	15 (5.9)	14 (5.8)	19 (7.5)
- **Encephalopathy/encephalitis**	12 (5.0)	22 (8.6)	19 (7.9)	15 (5.9)
- **Brain Tumor**	6 (2.5)	8 (3.1)	8 (3.3)	6 (2.4)
- **Other**	54 (22.5)	45 (17.6)	53 (21.9)	46 (18.1)
**ARDS etiology**[6]				
- **Pneumonia**	145 (47.1)	144 (48.0)	143 (45.7)	146 (49.5)
- **Sepsis**	90 (29.2)	78 (26.0)	86 (27.5)	82 (27.8)
- **Gastric content aspiration**	29 (9.4)	30 (10.0)	33 (10.5)	26 (8.8)
- **COVID-19**	15 (4.9)	10 (3.3)	13 (4.2)	12 (4.1)
- **Thoracic trauma**	10 (3.2)	11 (3.7)	12 (3.8)	9 (3.1)
- **Pancreatitis**	9 (2.9)	11 (3.7)	11 (0.3)	9 (3.1)
- **Cardiopulmonary bypass**	3 (1.0)	2 (0.7)	3 (1.0)	2 (0.7)
- **Non-thoracic trauma**	1 (0.3)	2 (0.7)	1 (0.3)	2 (0.7)
- **Smoke/toxin inhalation**	0 (0.0)	2 (0.7)	2 (0.6)	0 (0.0)
- **Near - drowning**	0 (0.0)	1 (0.3)	1 (0.3)	0 (0.0)
- **Other**	6 (1.9)	9 (3.0)	8 (2.6)	7 (2.4)
**Mode of ventilation**				
- **SIMV**	288 (29.8)	289 (29.9)	291 (30.1)	286 (29.7)
- **CPAP/ASB**	188 (19.5)	173 (17.9)	172 (17.8)	189 (19.6)
- **PS**	166 (17.2)	178 (18.4)	173 (17.9)	171 (17.7)
- **BIPAP**	124 (12.9)	121 (12.5)	127 (13.1)	118 (12.2)
- **PRVC**	101 (10.5)	94 (9.7)	100 (10.3)	95 (9.9)
- **PC**	58 (6.0)	73 (7.6)	65 (6.7)	66 (6.9)
- **APRV**	35 (3.6)	34 (3.5)	33 (3.4)	36 (3.7)
- **HFOV**	0 (0.0)	0 (0.0)	0 (0.0)	0 (0.0)
- **Other**	5 (0.5)	4 (0.4)	6 (0.6)	3 (0.3)
**Plateau Pressure (cmH_2O)**	22.0±6.1<br>n=390	21.7±6.1<br>n=379	22.2±5.9<br>n=389	21.5±6.3<br>n=380
**Driving Pressure (cmH_2O)**	13.7±5.5<br>n=379	13.3±5.2<br>n=371	13.9±5.0<br>n=380	13.2±5.6<br>n=370

*Data are presented as n (%). Percentages may not total 100 due to rounding

Abbreviations: RA = rheumatoid arthritis, OA = osteoarthritis, COPD = chronic obstructive pulmonary disease, ARDS = acute respiratory distress syndrome, CHF = congestive heart failure, MI = myocardial infarction, BMI = body mass index, TIA = transient ischaemic attack, GI = gastrointestinal, COPD = chronic obstructive pulmonary disease, COVID-19 = coronavirus disease, SIMV = synchronised intermittent mandatory ventilation, CPAP/ASB =continuous positive airways pressure/assisted spontaneous breathing, PS = pressure support, BIPAP = bilevel positive airway pressure, PRVC = pressure regulated volume control, PC = pressure control, APRV = airway pressure release ventilation, HFOV = high frequency oscillatory ventilation
[1]Back disease, spinal stenosis or severe chronic back pain; [2]Very hard of hearing, even with hearing aids; [3]Such as Multiple Sclerosis or Parkinson's Disease; [4]For example ulcer, hernia, reflux; [5]For example cataracts, glaucoma, macular degeneration). [6]Patients may have ≥1 ARDS etiology; % based on total number of reasons (ARDS etiology; 1, n=241 patients, 2, n=133 patients, 3, n=29 patients, 4, n=4 patients)

S5. Treatment after trial entry by comparison group

Table S5. Treatment after trial entry by comparison group

		Carbocisteine	No Carbocisteine	Hypertonic Saline	No Hypertonic Saline
		**n=965**	**n=966**	**n=967**	**n=964**
**No. of days on treatment (CS)[a]**	*Mean (95% CI)*	12.2 (11.7 to 12.7) n=964	n/a	12.2 (11.5 to 12.9) n=485	12.2 (11.5 to 12.9) n=479
**No. of days on treatment (HTS)[a]**	*Mean (95% CI)*	12.2 (11.5 to 12.9) n=485	11.7 (11.0 to 12.4) n=481	12.0 (11.4 to 12.5) n=966	n/a
**No. of days on treatment [a]**	*Mean (95% CI)*	12.2 (11.7 to 12.7) n=964	11.7 (11.0 to 12.4) n=481	12.0 (11.4 to 12.5) n=966	12.2 (11.5 to 12.9) n=479
**Treatment Compliance[#]**	**Compliant**	798 (85.0%)	405 (86.5%)	803 (85.8%)	400 (84.9%)
	**Not Compliant**	141 (15.0%)	63 (13.5%)	133 (14.2%)	71 (15.1%)
	**Compliance Rate (%)** *Mean (95% CI)*	94.0 (93.1 to 94.9) n=939	94.3 (93.0 to 95.7) n=468	94.2 (93.3 to 95.1) n=936	94.1 (92.7 to 95.4) n=471
**Non-trial Mucoactive Administration (Carbocisteine and Hypertonic Saline)**	**Number of patients with non-trial mucoactives administered at least once *(pts may have more than one mucoactive administered)***	45 (4.7%)	157 (16.3%)	41 (4.2%)	161 (16.7%)
	**Total number of days on non-trial mucoactive, per patient** *Mean (95% CI)*	6.4 (4.4 to 8.3) n=44	9.9 (8.2 to 11.6) n=156	7.2 (4.9 to 9.5) n=41	9.6 (8.0 to 11.3) n=159
	**Total number of days receiving full dose of non-trial mucoactive, per patient** *Mean (95% CI)*	7.5 (4.8 to 10.1) n=17	10.7 (8.6 to 12.8) n=93	7.8 (5.1 to 10.5) n=26	10.9 (8.7 to 13.1) n=84

		Carbocisteine	No Carbocisteine	Hypertonic Saline	No Hypertonic Saline
		n=965	n=966	n=967	n=964
Antibiotic Usage[a]	**Number of patients prescribed antibiotics at least once**<br>**Yes**	762 (79.0%)	771 (79.9%)	759 (78.6%)	774 (80.3%)
	**No** *(including uncertain)*	203 (21.0%)	194 (20.1%)	207 (21.4%)	190 (19.7%)
	**Number of days on antibiotic**<br>*Mean (95% CI)*	11.4 (10.7 to 12.1)<br>n=749	12.1 (11.2 to 12.9)<br>n=758	11.6 (10.8 to 12.4)<br>n=746	11.8 (11.1 to 12.6)<br>n=761
**Usual Airway Clearance Management**	**Number of sessions**<br>*Mean (95% CI)*	10.9 (10.3 to 11.5)	11.0 (10.4 to 11.7)	11.0 (10.4 to 11.6)	11.0 (10.3 to 11.6)
**Day 28 mortality**	**Dead**	112 (23.4%)	108 (22.5%)	108 (22.2%)	121 (25.0%)
	**Alive**	367 (76.6%)	373 (77.6%)	378 (77.8%)	364 (75.1%)
**Day 60 mortality**	**Dead**	125 (26.1%)	128 (26.6%)	120 (24.7%)	141 (29.1%)
	**Alive**	354 (73.9%)	353 (73.4%)	366 (75.3%)	344 (70.9%)
**6 month mortality**	**Dead**	135 (28.2%)	140 (29.1%)	141 (29.0%)	152 (31.3%)
	**Alive**	344 (71.8%)	341 (70.9%)	345 (71.0%)	333 68.7%)

Data are mean (95% CI) for continuous variables and no. (%) for categorical variables

[a]Number of days on treatment is irrespective of dose and/or treatment administration and is simply based on the time between treatment discontinuation and randomization

[#]Overall compliance defined as patients who receive ≥90% available doses. Carbocisteine administration compliance rate calculated as ((total doses- excluded doses)/(expected doses – 6))*100 where total doses = sum of all doses, excluded doses = sum of doses on day 1 and last day, expected doses = (total. no days*3), and expected doses excluding day 1 and last day = 2* 3 = 6. Hypertonic saline administration compliance rate calculated as ((total doses- excluded doses)/(expected doses – 8))*100 where total doses = sum of all doses, excluded doses = sum of doses on day 1 and last day, expected doses = (total. no days*4), and expected doses excluding day 1 and last day = 2* 4 = 8

S6. Primary outcome (intention to treat)

Table S6. Primary outcome (intention to treat)

**Primary Outcome[a] - duration of mechanical ventilation**		**Hypertonic Saline**		**Total**
		**No**	**Yes**	
**Carbocisteine**	**No** *Median (95% CI)*	172.7 (165.2 to 194.1) n=478 (Usual care)	172.9 (150.3 to 193.6) n=474 (Hypertonic Saline)	172.7 (165.2 to 190.4) n=952 (No Carbocisteine)
	**Yes** *Median (95% CI)*	176.2 (164.9 to 206.8) n=472 (Carbocisteine)	187.2 (166.0 to 209.5) n=479 (Hypertonic Saline plus Carbocisteine)	186.1 (168.3 to 196.6) n=951 (Carbocisteine)
**Total**	*Median (95% CI)*	174.3 (166.9 to 192.7) n=950 (no Hypertonic Saline)	184.5 (165.6 to 194.1) n=953 (Hypertonic Saline)	

**Duration of mechanical ventilation**	**Carbocisteine**	**No Carbocisteine**	**Hypertonic Saline**	**No Hypertonic Saline**
Median (95% CI)	186.1 (168.3 to 196.6) n=951 (Carbocisteine)	172.7 (165.2 to 190.4) n=952 (No Carbocisteine)	184.5 (165.6 to 194.1) n=953 (Hypertonic Saline)	174.3 (166.9 to 192.7) n=950 (no Hypertonic Saline)
**Hazard Ratio (95% CI)**	0.96 (0.87 to 1.05)		1.00 (0.91 to 1.10)	
**p-value[a,b]**	0.345		0.980	
**Hazard Ratio (95% CI)**	0.96 (0.88 to 1.05)		1.00 (0.91 to 1.10)	
**p-value[b]**	0.359		0.999	
**Sensitivity analysis – impact of interaction**				
**Hazard Ratio (95% CI)**	1.01 (0.83 to 1.22)			
**p-value[a,b]**	0.912			

Data are median (95% CI)

[a]adjusted for age and illness severity (APACHE II) and site included as a random effect

[b]from Cox-Proportional Hazard

Table S7. Post hoc pairwise comparisons between treatment groups

	Observed Median (IQR)	HR [a]	95% CI*
n=949	G1: 177.8 (89.9, 400.9), n=471<br>G4: 172.7 (87.0, 377.7), n=478	1.038	0.911 to 1.183
n=952	G2: 172.9 (80.4, 402.1), n=474<br>G4: 172.7 (87.0, 377.7), n=478	0.996	0.874 to 1.134
n=957	G3: 187.2 (81.7, 379.1), n=479<br>G4: 172.7 (87.0, 377.7), n=478	1.043	0.916 to 1.187
n=945	G1: 177.8 (89.9, 400.9), n=471<br>G2: 172.9 (80.4, 402.1), n=474	1.053	0.923 to 1.201

[a]Hazard ratios are from Cox proportional hazards models adjusted for age and APACHE II score with a shared frailty for site
HR = hazard ratio; CI = confidence interval
Group 1 – Carbocisteine; Group 2 – Hypertonic Saline; Group 3 - Carbocisteine and Hypertonic Saline; Group 4 – Usual care
Group 1 is the reference group therefore the HR is for Group 2 compared to Group 1
*The widths of confidence intervals have not been adjusted for multiplicity and cannot be used to infer treatment effects

Table S8. Complier Average Causal Effect analysis

Duration of mechanical ventilation	Carbocisteine	No Carbocisteine	Hypertonic Saline	No Hypertonic Saline
**CACE**				
**HR[a] (95% CI)**	0.91 (0.79 to 1.03)		0.97 (0.86 to 1.09)	

[a] Hazard ratios are from two-stage residual inclusion Cox proportional hazards models estimating the complier average causal effect, adjusted for age and APACHE II score, with shared frailty for site

*The widths of confidence intervals have not been adjusted for multiplicity and cannot be used to infer treatment effects

S9. Secondary analyses

Table S9. Primary outcome secondary analysis (Survivors)

**Primary Outcome[a] - duration of mechanical ventilation – Survivors***		**Hypertonic Saline**		**Total**
		**No**	**Yes**	
**Carbocisteine**	**No**<br>*Median (95% CI)*	169.3 (161.1 to 193.9)<br>n=375<br>(Usual care)	188.1 (155.5 to 214.0)<br>n=381<br>(Hypertonic Saline)	180.2 (165.1 to 193.3)<br>n=756<br>(No Carbocisteine)
	**Yes**<br>*Median (95% CI)*	168.6 (148.8 to 205.7)<br>n=374<br>(Carbocisteine)	193.4 (165.6 to 217.8)<br>n=386<br>(Carbocisteine plus Hypertonic Saline)	182.0 (165.6 to 207.3)<br>n=761<br>(Carbocisteine)
**Total**<br>*Median (95% CI)*		169.0 (164.2 to 191.0)<br>n=750<br>(No Hypertonic Saline)	188.7 (167.5 to 212.3)<br>n=767<br>(Hypertonic Saline)	

	**Carbocisteine**	**No Carbocisteine**	**Hypertonic Saline**	**No Hypertonic Saline**
Median (95% CI	182.0 (165.6 to 207.3)<br>n =761<br>(Carbocisteine)	180.2 (165.1 to 193.3)<br>n =756<br>(No Carbocisteine)	188.7 (167.5 to 212.3)<br>n=767<br>(HTS)	169.0 (164.2 to 191.0)<br>n=750<br>(No HTS)
**Hazard Ratio (95% CI)[a]**	0.94 (0.85 to 1.05)		0.95 to (0.86 to 1.06)	
**Hazard Ratio (95% CI)** $_{\text{Unadjusted}}$	0.95 (0.86 to 1.05)		0.95 (0.86 to 1.06)	

Data are median (95% confidence interval, CI)

[a]adjusted for age and illness severity (APACHE II) and site included as a random effect

[b]from Cox-Proportional Hazard

*The widths of confidence intervals have not been adjusted for multiplicity and cannot be used to infer treatment effects

S10. Secondary analyses

Table S10. Primary outcome secondary analysis (Non-survivors)

**Primary Outcome[a] - duration of mechanical ventilation – Non-survivors***		**Hypertonic Saline**		**Total**
		**No**	**Yes**	
**Carbocisteine**	**No** *Median (95% CI)*	188.0 (151.8 to 245.5) n =103 (Usual care)	153.6 (128.1 to 181.9) n =93 (Hypertonic Saline)	170.6 (148.1 to 193.9) n =196 (No Carbocisteine)
	**Yes** *Median (95% CI)*	198.9 (173.6 to 228.8) n =97 (Carbocisteine)	175.6 (137.8 to 196.1) n =93 (Carbocisteine plus Hypertonic Saline)	187.1 (169.1 to 206.5) n =190 (Carbocisteine)
**Total** *Median (95% CI*		193.7 (173.2 to 224.2) n =200 (No Hypertonic Saline)	169.6 (146.6 to 186.8) n =186 (Hypertonic Saline)	

	**Carbocisteine**	**No Carbocisteine**	**Hypertonic Saline**	**No Hypertonic Saline**
Median (95% CI)	187.1 (169.1 to 206.5) n =190 (Carbocisteine)	170.6 (148.1 to 193.9) n =196 (No Carbocisteine)	163.9 (146.6 to 186.8) n =186 (Hypertonic Saline)	193.7 (173.2 to 224.2) n =200 (No Hypertonic Saline)
**Hazard Ratio (95% CI)[c]**	1.03 (0.85 to 1.25)		1.39 (1.11 to 1.74)	
**Hazard Ratio (95% CI) Unadjusted**	1.02 (0.83 to 1.24)		1.37 (1.11 to 1.68)	

Data are median (95% confidence interval, CI)

[a]adjusted for age and illness severity (APACHE II) and site included as a random effect

[b]from Cox-Proportional Hazard

*The widths of confidence intervals have not been adjusted for multiplicity and cannot be used to infer treatment effects

S11. Subgroup analyses

Table S11. Primary outcome subgroup analyses

**Primary Outcome[a] - duration of mechanical ventilation (hours)**		**Carbocisteine vs No Carbocisteine**			**Hypertonic Saline vs No Hypertonic Saline**		
		**Carbocisteine**	**No Carbocisteine**	**HR (99% CI)**	**Hypertonic Saline**	**No Hypertonic Saline**	**HR (99% CI)**
**APACHE II quintiles[a*]**	**Q1** *Median (95% CI)*	164.5 (136.4 to 207.9) n=228	166.9 (141.2 to 188.8) n=241	0.99 (0.78 to 1.26)	164.3 (137.9 to 187.8) n=232	168.3 (141.4 to 196.4) n=237	1.01 (0.79 to 1.29)
	**Q2** *Median (95% CI)*	192.9 (154.1 to 237.4) n=176	148.8 (119.3 to 194.0) n=161	0.85 (0.64 to 1.14)	169.7 (141.2 to 225.2) n=171	174.2 (141.2 to 213.0) n=166	0.92 (0.69 to 1.22)
	**Q3** *Median (95% CI)*	187.4 (162.5 to 236.6) n=171	214.2 (170.0 to 256.5) n=168	0.94 (0.71 to 1.26)	214.0 (172.3 to 244.4) n=165	187.3 (165.2 to 252.4) n=174	0.97 (0.73 to 1.30)
	**Q4** *Median (95% CI)*	176.4 (149.4 to 198.1) n=188	177.1 (147.4 to 236.2) n=191	1.03 (0.79 to 1.35)	186.6 (163.7 to 215.4) n=204	169.3 (142.2 to 210.7) n=175	1.05 (0.80 to 1.38)
	**Q5** *Median (95% CI)*	196.6 (168.4 to 226.8) n=187	170.1 (146.7 to 209.6) n=191	0.96 (0.73 to 1.26)	177.1 (146.4 to 218.3) n=181	193.0 (167.4 to 216.9) n=197	1.05 (0.80 to 1.38)
**PaO_2/FiO_2 ratio[b*]**	**Mild** *Median (95% CI)*	165.8 (142.7 to 186.7) n=309	145.9 (120.5 to 169.2) n=298	0.9 2(0.74, 1.14)	153.9 (134.9 to 170.9) n=304	161.1 (121.9 to 177.8) n=303	1.03 (0.83 to 1.27)

Primary Outcome[a] - duration of mechanical ventilation (hours)		Carbocisteine vs No Carbocisteine			Hypertonic Saline vs No Hypertonic Saline		
		Carbocisteine	No Carbocisteine	HR (99% CI)	Hypertonic Saline	No Hypertonic Saline	HR (99% CI)
	**Moderate** *Median (95% CI)*	208.8 (182.0 to 238.2) n=459	193.0 (169.9 to 217.3) n=467	0.92 (0.78 to 1.10)	196.1 (173.8 to 235.5) n=467	196.0 (173.2 to 226.6) n=459	1.01 (0.85 to 1.20)
	**Severe** *Median (95% CI)*	180.1 (148.8 to 220.5) n=170	187.1 (154.9 to 214.0) n=177	1.08 (0.81 to 1.43)	191.1 (161.3 to 232.3) n=172	170.1 (147.6 to 201.7) n=175	0.92 (0.69 to 1.22)
**Pre-existing chronic respiratory condition***	**Yes** *Median (95% CI)*	197.9 (167.0 to 236.0) n=194	168.9 (141.5 to 192.6) n=208	0.78 (0.60 to 1.01)	184.6 (144.4 to 206.6) n=213	189.7 (164.4 to 210.0) n=189	1.05 (0.81 to 1.37)
	**No** *Median (95% CI)*	176.4 (165.6 to 194.1) n=756	174.7 (165.5 to 194.4) n=744	1.01 (0.88 to 1.16)	184.2 (164.5 to 196.2) n=740	173.0 (166.0 to 192.1) n=760	0.99 (0.86 to 1.13)
**Neurological diagnosis***	**Yes** *Median (95% CI)*	186.9 (146.3 to 208.3) n=234	190.0 (165.5 to 237.1) n=249	1.06 (0.83 to 1.35)	186.3 (153.4 to 212.1) n=235	192.8 (165.0 to 240.7) n=248	1.07 (0.84 to 1.36)
	**No** *Median (95% CI)*	184.5 (167.6 to 206.6) n=717	169.7 (159.2 to 189.1) n=702	0.92 (0.80 to 1.06)	182.2 (164.5 to 196.5) n=718	172.3 (165.5 to 189.8) n=701	0.98 (0.85 to 1.12)
**Receiving antibiotics for pulmonary infection***	**Yes** *Median (95% CI)*	174.4 (162.8 to 194.0) n=679	172.5 (164.4 to 192.1) n=685	1.00 (0.87 to 1.15)	168.9 (150.7 to 190.4) n=676	184.7 (168.5 to 196.1) n=688	1.04 (0.90 to 1.20)
	**No** *Median (95% CI)*	202.9 (169.7 to 229.8) n=272	175.0 (142.0 to 208.5) n=267	0.86 (0.68 to 1.08)	209.3 (180.8 to 236.9) n=277	167.0 (145.7 to 204.7) n=262	0.91 (0.72 to 1.14)
**Admission diagnostic**	**Pulmonary**	173.2 (147.9 to 196.6)	193.5 (166.4 to 226.5)	1.00 (0.80 to 1.26)	189.3 (164.5 to 215.1)	173.9 (150.9 to 208.6)	0.96 (0.77 to 1.21)

Primary Outcome[a] - duration of mechanical ventilation (hours)		Carbocisteine vs No Carbocisteine			Hypertonic Saline vs No Hypertonic Saline		
		Carbocisteine	No Carbocisteine	HR (99% CI)	Hypertonic Saline	No Hypertonic Saline	HR (99% CI)
categories (pulmonary/ non-pulmonary)8	*Median (95% CI)*	n=287	n=260		n=283	n=264	
	**Non-Pulmonary** *Median (95% CI)*	181.9 (166.4 to 207.2) n=633	169.2 (156.8 to 188.4) n=662	0.96 (0.83 to 1.11)	174.4 (154.8 to 191.8) n=635	173.0 (166.3 to 193.4) n=660	1.02 (0.88 to 1.18)
**Compliant[c,d*]**	**Yes** *Median (95% CI)*	192.7 (174.7 to 209.7) n=786	190.3 (164.4 to 218.8) n=399	0.98 (0.83 to 1.15)	191.8 (175.7 to 212.5) n=792	189.3 (168.7 to 216.3) n=393	0.96 (0.82 to 1.13)
	**No** *Median (95% CI)*	147.9 (115.0 to 209.9) n=139	121.0 (75.2 to 169.5) n=63	0.69 (0.46 to 1.04)	142.0 (101.5 to 183.5) n=131	130.4 (94.7 to 210.3) n=71	1.39 (0.94 to 2.06)
**Contaminated[e*]**	**Yes** *Median (95% CI)*	309.9 (233.0 to 447.5) n=31	292.9 (237.3 to 349.4) n=130	1.19 (0.76 to 1.89)	308.8 (210.4 to 430.5) n=43	292.9 (242.9 to 338.0) n=136	1.06 (0.67 to 1.69)
	**No** *Median (95% CI)*	175.7 (166.2 to 193.9) n=919	165.5 (146.5 to 173.2) n=821	0.90 (0.79 to 1.02)	177.1 (164.3 to 191.5) n=910	166.6 (152.2 to 173.8) n=814	0.95 (0.84 to 1.08)

Mean (95% CI) presented for each subgroup

[b]PaO_2/FiO_2 ratio 201-300mmHg or 26.7-39.9kPa (Mild); 101-200mmHg or 13.3-26.6kPa (Moderate), <100mmHg or <13.3kPa (Severe)

[c]Patients who received ≥90% available doses were considered as compliant

[d]No values available for patients receiving usual care only (no mucoactive)

[e]Contaminated was considered as any episode of non-trial mucoactive (NTM, either carbocisteine and/or hypertonic saline) administration that had ≥24 hour duration (regardless of when the NTM commenced) i.e. this means the patient receives >4 individual doses of hypertonic saline and/or >3 individual doses carbocisteine (assuming that beyond Day 0, a 24 hour dose = 4 hypertonic saline, 3 carbocisteine). Any episode that stopped on day of randomization did not count as contamination (i.e. NTM that were within a 24 hour period around the timepoint of randomization). Any patient with two or more episodes of NTM lasting <24 hours counted as contamination

*The widths of confidence intervals have not been adjusted for multiplicity and cannot be used to infer treatment effects

S12. Sensitivity analyses

Table S12. Primary outcome sensitivity analyses

Primary Outcome[a] - duration of mechanical ventilation (hours)	Carbocisteine	No Carbocisteine	Hypertonic Saline	No Hypertonic Saline
**Competing risk of death[b*]**				
**Sub Hazard Ratio (95% CI)**	0.97 (0.88 to 1.07)		1.02 (0.91 to 1.14)	
**Impact of contamination[c] between interventions (i.e. excluding contaminated patients)**				
Median (95% CI)	175.7 (166.2 to 193.9) n=909	165.5 (146.5 to 173.2) n=815	177.1 (164.3 to 191.5) n=911	166.6 (152.2 to 173.8) n=813
**Hazard Ratio (95% CI)**	0.90 (0.82 to 0.99)		0.96 (0.87 to 1.05)	
**Impact of compliance[d] between interventions (i.e. excluding non-compliant patients)***				
Median (95% CI)	192.7 (174.7 to 209.7) n=786	190.3 (164.4 to 218.8) n=399	191.8 (175.7 to 212.5) n=792	189.3 (168.7 to 216.3) n=393
**Hazard Ratio (95% CI)**	0.98 (0.87 to 1.11)		0.97 (0.85 to 1.10)	
**Excluding patients not receiving invasive mechanical ventilation at the point of randomization[e*]**				
Median (95% CI)	186.5 (168.6 to 197.6) n=945	174.4 (165.6 to 191.2) n=947	186.1 (166.8 to 195.2) n=947	175.0 (167.1 to 193.6) n=945
**Hazard Ratio (95% CI)**	0.96 (0.87 to 1.05)		1.00 (0.92 to 1.10)	

[a]Intention-to-treat population

[b]Sub Hazard ratio (SHR) with 95% CI (confidence interval)

[c]Contaminated was considered as any episode of non-trial mucoactive (NTM, clarified to be either carbocisteine and/or hypertonic saline) administration that had ≥24 hour duration (regardless of when the NTM commenced) i.e. this means the patient receives >4 individual doses of hypertonic saline and/or >3 individual doses carbocisteine (assuming that beyond Day 0, a 24 hour dose = 4 hypertonic saline, 3 carbocisteine). Any episode that stopped on day of randomization did not count as contamination (i.e. NTM that were within a 24 hour period around the timepoint of randomization). Any patient with two or more episodes of NTM lasting <24 hours counted as contamination.

[d]Patients who received ≥90% available doses were considered as compliant. No values available for patients receiving usual care only (no mucoactive)

[e]Patients identified post randomization as having not been on invasive mechanical ventilation at the point of randomization (n=11)

*The widths of confidence intervals have not been adjusted for multiplicity and cannot be used to infer treatment effects

S13. Adverse event reporting

Table S13. Adverse event reporting

		Number of Events[a]		Number of Patients[b]		Number of Events[a]		Number of Patients[b]	
		Carbocisteine	No Carbocisteine	Carbocisteine	No Carbocisteine	Hypertonic Saline	No Hypertonic Saline	Hypertonic Saline	No Hypertonic Saline
		n=1	n=0	n=965	n=966	n=1	n=0	n=967	n=964
SAEs and SUSAR	Total SAES	1 (100.0%)	0 (0.0%)	1 (0.1%)	0 (0.0%)	1 (100.0%)	0 (0.0%)	1 (0.1%)	0 (0.0%)
	Related to study drug	1 (100.0%)	0 (0.0%)	1 (0.1%)	0 (0.0%)	1 (100.0%)	0 (0.0%)	1 (0.1%)	0 (0.0%)
	Related to study drug and unexpected	0 (0.0%)	0 (0.0%)	0 (0.0%)	0 (0.0%)	0 (0.0%)	0 (0.0%)	0 (0.0%)	0 (0.0%)
	Total Deaths	0 (0.0%)	0 (0.0%)	0 (0.0%)	0 (0.0%)	0 (0.0%)	0 (0.0%)	0 (0.0%)	0 (0.0%)
SARs	Gastro-intestinal	1 (100.0%)	0 (0.0%)	1 (0.1%)	0 (0.0%)	1 (100.0%)	0 (0.0%)	1 (0.1%)	0 (0.0%)
SUSARs	Gastro-intestinal	0 (0.0%)	0 (0.0%)	0 (0.0%)	0 (0.0%)	0 (0.0%)	0 (0.0%)	0 (0.0%)	0 (0.0%)

[a]percentages calculated using total number of events within each group

[b]percentages calculated using total number of patients within each group

Abbreviations: SAE = serious adverse event; SUSAR = suspected unexpected serious adverse reaction; SAR = serious adverse reaction

REFERENCES

1. Connolly B, Dickson N, Agus A, et al. Effectiveness of mucoactives (carbocisteine and hypertonic saline) in addition to usual airway clearance management with usual airway clearance management alone in acute respiratory failure (MARCH): study protocol for a multi-centre 2x2 factorial, randomised, controlled, open-label, Phase 3, pragmatic, clinical and cost-effectiveness trial with internal pilot [version 1; peer review: 2 approved]. NIHR Open Research 2025;5(30).
2. Connolly B, Barclay M, Blackwood B, et al. Airway clearance techniques and use of mucoactive agents for adult critically ill patients with acute respiratory failure: a qualitative study exploring UK physiotherapy practice. Physiotherapy 2020;108:78-87.
3. Case Mix Programme Public Report 2022–23 Summary Statistics. Intensive Care National Audit and Research Centre (ICNARC). Available at https://www.icnarc.org/reports/case-mix-programme-public-report-2022-23/. Accessed 28/02/2026. 2024.
4. An evidence summary of health inequalities in older populations in coastal and rural areas. Public Health England. Available at https://www.gov.uk/government/publications/health-inequalities-in-ageing-in-rural-and-coastal-areas. Accessed 10/02/2026. 2019.
5. Office for National Statistics Rural-Urban Classification - Web map for Rural Urban Classification (RUC) of Local Authority District Areas (LADs), England and Wales, 2024. Available at https://geoportal.statistics.gov.uk/maps/3b274939bfb84a97867ce0531973c243. Accessed 10/02/2026. 2024.
6. Scottish Government Urban Rural Classification 2022. Available at https://www.gov.scot/publications/scottish-government-urban-rural-classification-2022/pages/overview/. Accessed 10/02/2026. 2024.

7. Northern Ireland Statistics and Research Agency and Department of Agriculture, Environment and Rural Affairs Rural Data Hub. Available at https://daera-sasb.github.io/Rural-Data-Hub/. Accessed 10/02/2026. 2026.

8. The PEPTIC Investigators for the Australian New Zealand Intensive Care Society Clinical Trials Group, Alberta Health Services Critical Care Strategic Clinical Network, the Irish Critical Care Trials Group. Effect of Stress Ulcer Prophylaxis With Proton Pump Inhibitors vs Histamine-2 Receptor Blockers on In-Hospital Mortality Among ICU Patients Receiving Invasive Mechanical Ventilation: The PEPTIC Randomized Clinical Trial. JAMA 2020;323(7):616-626.

9. van Meenen DMP, van der Hoeven SM, Binnekade JM, et al. Effect of On-Demand vs Routine Nebulization of Acetylcysteine With Salbutamol on Ventilator-Free Days in Intensive Care Unit Patients Receiving Invasive Ventilation: A Randomized Clinical TrialEffects of On-Demand vs Routine Nebulization of Acetylcysteine and Salbutamol on Ventilation Duration in ICU PatientsEffects of On-Demand vs Routine Nebulization of Acetylcysteine and Salbutamol on Ventilation Duration in ICU Patients. JAMA 2018;319(10):993-1001.

10. Dixon B, Smith RJ, Campbell DJ, et al. Nebulised heparin for patients with or at risk of acute respiratory distress syndrome: a multicentre, randomised, double-blind, placebo-controlled phase 3 trial. Lancet Respir Med 2021;9(4):360-372.
